# Embodied Rationality Through Game Theoretic Glasses: An Empirical Point of Contact

**DOI:** 10.3389/fpsyg.2022.815691

**Published:** 2022-04-11

**Authors:** Sébastien Lerique

**Affiliations:** Embodied Cognitive Science Unit, Okinawa Institute of Science and Technology Graduate University, Okinawa, Japan

**Keywords:** Team Rationality, Perceptual Crossing, Game Theory, Assurance game, Participatory Sense-Making, social awareness, Linguistic Bodies

## Abstract

The conceptual foundations, features, and scope of the notion of rationality are increasingly being affected by developments in embodied cognitive science. This article starts from the idea of embodied rationality, and aims to develop a frame in which a debate with the classical, possibly bounded, notion of rationality-as-consistency can take place. To this end, I develop a game theoretic description of a real time interaction setup in which participants' behaviors can be used to compare the enactive approach, which underlies embodied rationality, with game theoretic approaches to human interaction. The Perceptual Crossing Paradigm is a minimal interaction interface where two participants each control an avatar on a shared virtual line, and are tasked with cooperatively finding each other among distractor objects. It is well known that the best performance on this task is obtained when both participants let their movements coordinate with the objects they encounter, which they do without any prior knowledge of efficient interaction strategies in the system. A game theoretic model of this paradigm shows that this task can be described as an Assurance game, which allows for comparing game theoretical approaches and the enactive approach on two main fronts. First, accounting for the ability of participants to interactively solve the Assurance game; second, accounting for the evolution of choice landscapes resulting from evolving normative realms in the task. Similarly to the series of paradoxes which have fueled debates in economics in the past century, this analysis aims to serve as an interpretation testbed which can fuel the current debate on rationality.

## 1. Introduction

The conceptual foundations, features, and scope of the notion of rationality are increasingly being affected by developments in embodied cognitive science. This article starts from the idea of Embodied Rationality (Gallagher, 2018; Rolla, 2021) which, among the array of proposals bringing embodied cognition and rationality together, stands out with the following features (Petracca, 2021): (1) it is the most radical, both philosophically and in terms of its departure from Simon's original bounded rationality (Simon, 1956); (2) no empirical studies have yet been developed to support, falsify, or otherwise empirically distinguish it from other approaches—so far, the case for embodied rationality has been made at the conceptual and philosophical levels; (3) it connects with two issues that render it relevant across most domains in which rationality is currently discussed, namely, the scales of agency, and the dialectical evolution of normative realms.

Indeed, cognitively inspired modifications to the notion of rationality have traditionally entered the debate under the rubric of bounded rationality, separated in two different strands (Ross, 2014). On one side the psychology-driven tradition, which has convincingly shown the inadequacy of modeling an agent as capable of perfect predictions obtained using boundless resources. This tradition inherits from Simon's bounded rationality (Simon, 1956) and Gigerenzer's ecological rationality (Gigerenzer and Selten, 2002), and conceives rationality as successful adaptation to real-world tasks and situations. On the other side the economics tradition, interested in modeling collective behavior such as markets at the aggregate level, discusses rationality in terms of consistency between preferences, decision, and action. While this normative framework was originally developed for the individual level, underlying Rational Choice Theory, and further used by Kahneman and Tversky as the reference against which cognitive distortion, risk aversion and framing effects were evaluated (Kahneman, 2003), Becker (1962) argued early on that models of collective behavior need not make strong assumptions on the rationality or irrationality of individual agents: for results at the collective scale, it makes sense to approximate away from the details of psychological processes which may cancel each other out. Rubinstein (1998)'s seminal work makes a similar move for individual-level bounded rationality, providing a case-by-case evaluation of the relevance and effects of bounded mechanisms in models of collective behavior.

While notions of rationality have long been fragmented and debated, this conceptual divide seems to underpin the surprising idea that no matter the breadth of phenomena observed in psychology and behavioral economics, effects can be abstracted away or selectively added to otherwise unaffected premises of models of collective behavior. Infante et al. (2016), for instance, show that behavioral economists have largely adopted a dualist model of economic agents made of a rational core inside a psychological shell: the preferences of the shell can be revealed by traditional field experiments, but must then be purified in order to reveal the true, stable preferences of the rational core, which can therefore be used in economic models. At this point it is worth noticing the role that underlying metaphors of the mind play in the debate. Petracca (2021) groups the range of bounded rationality approaches into four, increasingly radical notions (Embodied Bounded Rationality, Body Rationality, Extended Rationality, and Embodied Rationality). The first three, which together cover the bounded rationality approaches presented above, remain broadly compatible with the computational metaphor of mind, albeit with increasing constraints^1^. This persistence of computationalist roots is likely to have played a role in the sedimentation of this conceptual divide: a set of models compatible with a qualified computational metaphor of mind, as the majority of bounded rationality approaches seem to be, can more easily be approximated as variations of a general set of premises (the ones underlying rationality-as-consistency), than an epistemologically more varied set of models.

Thus, by explicitly dropping computationalism and representations, and calling for a broader redefinition of rationality, embodied rationality (Gallagher, 2018; Rolla, 2021) provides a genuinely new element in the debate. Indeed, while the lineage of embodied rationality makes it directly relevant to the adaptive tradition of bounded rationality (see again Petracca, 2021), it would be a mistake to consider that modeling aggregate behavior under embodied rationality assumptions can be done similarly to, and with the same abstractions as, models built on classical bounded rationality-as-consistency^2^. Gallagher et al. (2019) and Petracca and Gallagher (2020), for instance, propose of view of markets as economic cognitive institutions, whereby “what is distributed in the market is not only information but also artifacts, routines, practices, social interactions, and affordances” (Petracca and Gallagher, 2020, p. 15), all “resources” which contribute to, and are affected by, the scales at which agency operates, the scaffolding of cognition and of interactions, autonomy, and ultimately becoming.

However, in order to trigger such a broad reevaluation of the abstractions underlying economic models, and advance to a workable understanding of the co-constitution of minds and collective behavior (markets seen as cognitive institutions being a case in point; Rizzello and Turvani, 2000 and Gallagher et al., 2019, p. 16), this argument also needs to be made at the level of models. This article aims to develop a frame in which such a debate can take place, that is, an empirical, model-friendly point of contact between embodied rationality and the classical, possibly bounded, notion of rationality-as-consistency. Similarly to the series of paradoxes which have fueled the debate on rationality in the past century, this point of contact aims to fuel the current debate by serving as an interpretation testbed.

I propose to do this by providing a new, game theoretic description of a well-studied sensorimotor interaction setup known as the Perceptual Crossing Paradigm (PCP). Through a series of lightweight approximations and empirically grounded assumptions, I will show that participants in recent versions of the PCP face a variation of the *Assurance game*. This game can be seen as a team-centered version of the well-known Prisoner's Dilemma (Ross, 2021), and is known for eliciting behaviors which standard Game Theory cannot account for. Instead, accounting for behaviors in the standard Assurance game using the classical notion of rationality-as-consistency requires articulating rationality with different scales of agency, a move which is made possible in two different ways by Team Reasoning and Conditional Game Theory (Sugden, 1993; Bacharach et al., 2006; Stirling, 2012; Hofmeyr and Ross, 2019). Since Embodied Rationality is directly compatible with the standard, enactive account of PCP, the identification of game theoretic structures in PCP provides a common empirical testbed for Embodied Rationality, Team Reasoning and Conditional Game Theory to compete for accounts of well-established PCP results.

I will discuss how both Team Reasoning and Conditional Game Theory successfully account for the different scales of agency at play in PCP, and will conclude by focusing on a process which has not yet been usefully formalized: the emergence and evolution of normative realms, and the resulting evolution of the strategies landscape. I contend that this use of the PCP, bringing such different approaches to rationality within arm's reach of each other, opens a path for refining our views of rationality in a way that can change the overall division of labor in modeling both individual behavior and collective behavior such as markets.

### 1.1. Relevant Works

**Figure 1:**
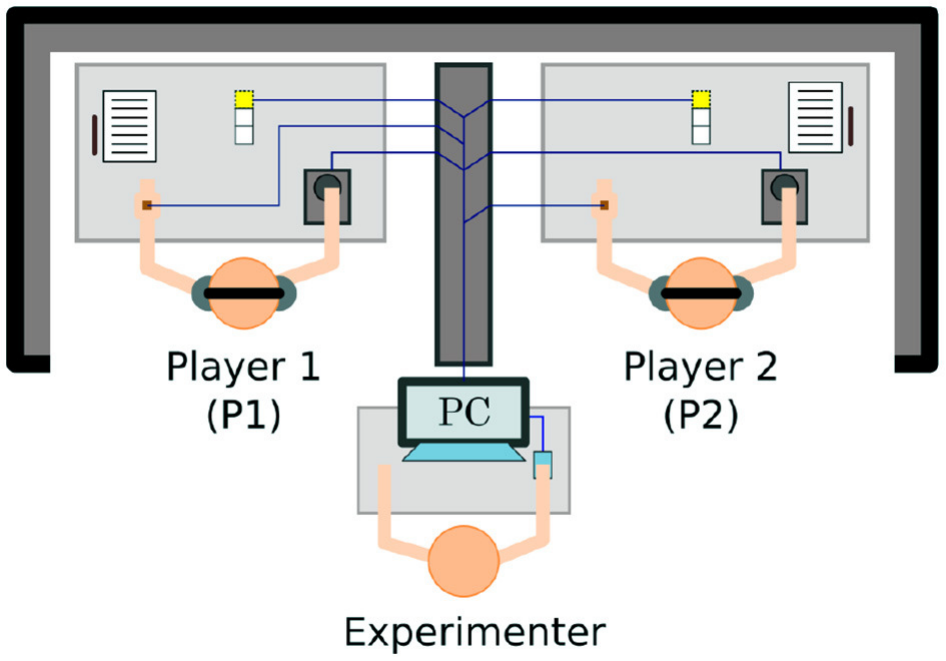
Participants and experimenter in the Perceptual Crossing Paradigm. Copied from Froese et al. (2014a) under CC BY 3.0.

**Figure 2:**
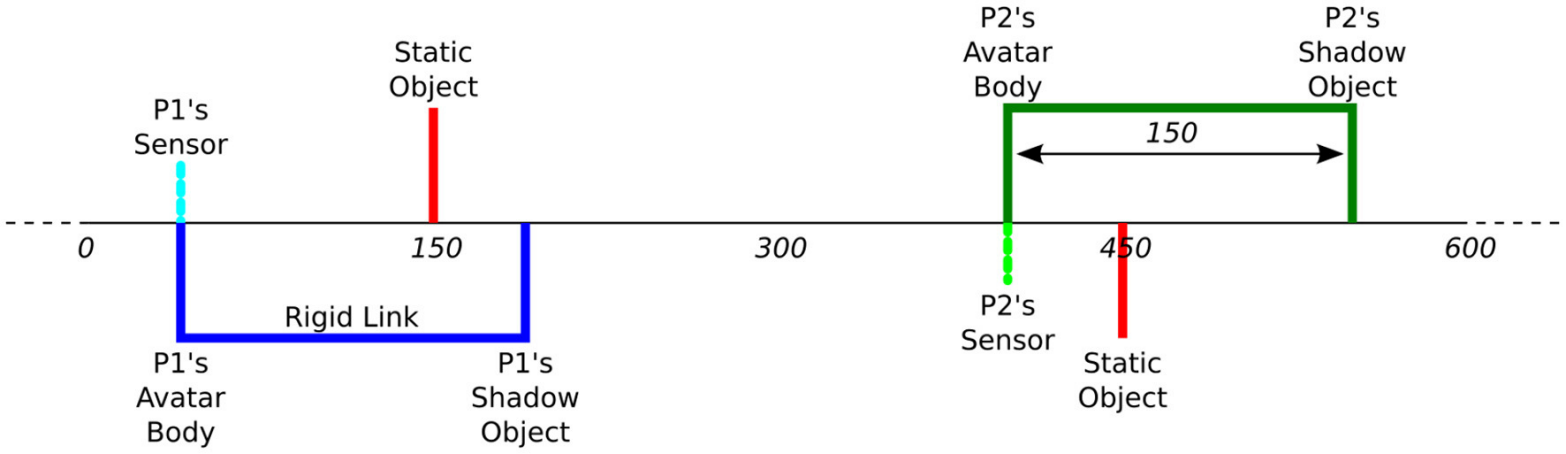
Virtual space in the Perceptual Crossing Paradigm, including the participants' avatars, static objects, and shadow objects. Note that each participant can feel only one static object, as indicated by the positioning of their sensor. Copied from Froese et al. (2014a) under CC BY 3.0.

The Perceptual Crossing Paradigm is first introduced by Auvray et al. (2009) as a new approach to the classic TV-mediated mother-infant interaction paradigm of Trevarthen and colleagues (Murray and Trevarthen, 1985, 1986; Nadel et al., 1999; Soussignan et al., 2006). The setup provides a minimal interaction interface where two participants each control an avatar on a shared virtual line. Each participant is given a device to move their avatar (often using a marble computer mouse) and receive haptic feedback through mechanical vibration. Using this device, each participant can move their avatar to explore different objects on the shared virtual line: a static object, the other participant's avatar, and a shadow object that mirrors the other participant's movements at a fixed distance. The line and objects (including the avatars) are invisible, but touching any object on the line (including the other person's avatar) is felt as mechanical vibration on the participant's device. Each participant is tasked with finding the other participant's avatar and clicking on it; the difficulty is therefore to distinguish between the other avatar and its shadow. Figures 1, 2 illustrate the experimental setup and virtual space.

The main interest in the initial version of this setup (Auvray et al., 2009), aside from its simplicity, lies in the fact that participants solve the task collectively but not individually. On one side, participants do not seem able to individually distinguish between avatar and shadow, and on the other the final number of clicks on the other avatar is higher than on the shadow. Success is attributable to the inherent stability of avatar-to-avatar interactions, that is, to a property of the dyadic dynamics that creates more opportunities to click on the avatar than on the shadow. The versatility of the setup has led to a decade of profuse study of the conditions influencing participants' behavior and performance on the task, and in particular the conditions that enable participants to develop a sense of social presence; I review these works in the next section. As a result, the setup has established itself as a major tool for exploring sensorimotor-based interaction dynamics, alongside other setups studying coordinated behavior and cooperation-based performance (Reed et al., 2006; Kelso, 2008; Nordham et al., 2018).

The theoretical understanding of PCP results mainly relies on the developments of Participatory Sense-Making in the context of the Enactive Approach (De Jaegher and Di Paolo, 2007; Froese and Di Paolo, 2011). Participatory Sense-Making describes the enactment of systems of multiple autonomous agents which go from individually regulating their interaction with their environment with respect to their own norms and identity (sense-making), to coordinated regulation, with other autonomous systems, of their interactions with their environment (participatory sense-making). As a result, “individual sense-making processes are affected and new domains of social sense-making can be generated that were not available to each individual on her own” (De Jaegher and Di Paolo, 2007, p. 497). It is important to note that this notion does not presuppose any social perception or pro-sociality. Auvray et al. (2009)'s initial version of the PCP is therefore a paradigmatic example of Participatory Sense-Making. When such interactions include a social component proper, they transition from Participatory Sense-Making to Social Agency, that is *co-regulation* of agents' interactions with their environment, that is of their sense-making (Di Paolo et al., 2018, p. 145). Social Agency obtains when participants in an interaction not only affect each other's environments (and thereby the conditions of their sense-making), but directly participate in each other's regulation of interaction with the environment, that is in each other's sense-making. As we will see, the more recent versions of PCP which were designed to elicit Social Agency are well-understood with this tooling, and exhibit other characteristic features of the Linguistic Bodies approach (Di Paolo et al., 2018): the existence of partial acts, and the dialectical dynamics of meaning due to evolving tensions between individual and interactive levels of normativity.

Drawing a link between Participatory Sense-Making and Linguistic Bodies on one side, and notions of rationality on the other, may seem challenging at first. On the enactive side, Embodied Rationality and Radically Enactive Rationality (Gallagher, 2018; Rolla, 2021) develop ways of thinking about rationality rooted in bodily performance. Future work will hopefully integrate the idea of rationality under enactivist hermeneutics with the Linguistic Bodies approach, accounting for the emergence of shared realms of rationality similarly to languaging. While such an integration has not yet been fleshed out, from here on I will take Embodied Rationality as the main notion of rationality associated with the enactive approach, and therefore with Participatory Sense-Making and the Linguistic Bodies approach.

Starting from the other side of the crevasse, Game Theory initially seems to be the obvious tool for analysing interdependent dyadic behavior in the rationality-as-consistency framework, and some attempts have been made to apply it to the study of coordinated joint action (Engemann et al., 2012). However, missing in game theory is the capacity to think about rationality at the level of the dyad, as can be seen in its failure to account for empirical results on the Hi-Lo game or the Assurance game (Ross, 2021). For this task, the most promising approaches are, on one side, Team Rationality, and on the other, the extension of Game Theory into Conditional Game Theory.

Robert Sugden seems to have been the first to argue that rationality at the level of a team is worth thinking about in the context of games and economic models. Criticizing approaches such as Schelling's theory of focal points (Schelling, 1960) for introducing external factors instead of expanding the notion of rationality itself, Sugden proposed that some games should be analyzed by asking how people rationally think when considering themselves part of a team (Sugden, 1993, 2003; Bacharach et al., 2006). The approach allows for solutions to standard problems such as the Hi-Lo game and the Assurance game which will reappear throughout this paper. It also dovetails with a broader proposal for a new form of normative economics built on the idea of sets of mutually acceptable market opportunities, instead of individual preferences, thus avoiding the common normative economics pitfall of considering agents mistaken in their unreliable preferences (Sugden, 2018).

A second approach to team phenomena is found in the recent extension of Game Theory into Conditional Game Theory (Stirling, 2012). This approach provides a framework for modeling situations where the preferences of some agents depend on the preferences of other agents. *Preference* conditioning, modeled over an acyclic network of influences between agents, goes beyond the simple interdependence of *choices* that is common in traditional Game Theory. Indeed, an agent's preferences are allowed to change depending on the preferences of influencing agents, after which choices are then made. This formalism also provides solutions to the Hi-Lo and Assurance games, while remaining compatible with traditional Game Theory in non-conditional situations.

While so far the two approaches seem to peacefully coexist (e.g., Lecouteux, 2018; Stirling and Tummolini, 2018; Ross, 2021), Hofmeyr and Ross (2019) correctly note that Conditional Game Theory provides a more general formalism which can also be used in cases of non-aligned groups. However, this observation misses Sugden's broader project of developing a form of normative economics which, by decoupling preferences from opportunities, need not bracket away the results of behavioral economics (Sugden, 2019). The debate is far from over, as the recent extension of Conditional Game Theory to cyclical influence networks (Stirling, 2019) introduces the proposal to the realm of (for now Markovian) stochastic processes. While beyond the scope of this article, this may in turn have relevance for a discussion with the heavily dynamical Linguistic Bodies approach.

How (in)compatible could Embodied Rationality, Team Rationality and Conditional Game Theory be, were they to find empirical applications in which to compare them? What notion of rationality would emerge from a beneficial exchange between these three theories? These are the two questions that I aim to bring into reach by looking at possible game theoretic structures in the PCP. I now start by reviewing previous studies and established results.

## 2. The Perceptual Crossing Paradigm

### 2.1. Experimental Setup

Let us first name the participants in a PCP experiment: Alice and Bob. Recall that for both Alice and Bob, the virtual line is populated with three objects:

1. a static object, whose position is fixed and does not move throughout the whole experiment; there is one static object per participant, and each participant can only feel their own static object;
2. the other avatar, which moves along the line as controlled by the other participant; when Alice and Bob's avatar are touching each other, both Alice and Bob receive haptic feedback;
3. a copy of the other participant's avatar, which is maintained at a fixed distance of their avatar, mirroring its movements; when Alice touches Bob's shadow avatar, Alice receives feedback and Bob does not (and reciprocally when Bob touches Alice's shadow).

The setup can be seen as a toy model for common interaction situations, for instance mutual eye gaze. In this analogy, the avatar-touches-avatar interaction has a similar structure to two people looking mutually at each other in the eyes, whereas the avatar-touches-shadow interaction is analogous to looking at someone who is looking away.

In this setup however, participants are only informed that there is a static object, a moving object, and the other person's avatar. Participants do not know, therefore, that the moving object that is not the other avatar is in fact mirroring the movements of the other participant. The setup is therefore closer to the mother-infant TV-mediated interaction setup introduced by Trevarthen (Murray and Trevarthen, 1985, 1986; Nadel et al., 1999; Soussignan et al., 2006), from which it was originally inspired.

### 2.2. Success Is Joint

Each participant is then tasked with finding the other participant's avatar in the virtual space, and clicking on it when they believe they have found it. In the original version introduced by Auvray et al. (2009), participants are trained in specific situations, and then have 15 min with short breaks to explore the space and interact, clicking as many times as they see fit.

Initially, the main interest in this setup is the combination of collective success and individual failure in solving the task. On one side, participants do not seem able to individually distinguish between avatar and shadow: the probability that they will click after an encounter with the avatar is not significantly different from the probability of clicking after an interaction with the shadow. Yet the final number of clicks on the other avatar is higher than on the shadow. The reason is that encounters with the other avatar are more frequent, due to a higher stability of the interaction: when the two avatars touch each other, both participants will come back on their steps and oscillate around each other; whereas when an avatar is touching a shadow, the other participant receives no feedback relating to this contact, and will therefore not engage in maintaining the interaction. As Auvray et al. (2009) put it: “If the participants succeeded in the perceptual task, it is essentially because they succeeded in situating their avatars in front of each other.” The setup therefore elicits success in a minimal task which can only be explained by the dynamics at the level of the dyad.

### 2.3. Social Awareness and Turn-Taking

The years following this work then chiefly focused on the question of what this behavior elicits about social cognition (Di Paolo et al., 2008; De Jaegher et al., 2010; Lenay et al., 2011; Auvray and Rohde, 2012; Froese et al., 2012; Lenay, 2012), and what minimal change to the setup could test the strong interpretation according to which social cognition can be partly constituted by social interaction (Michael, 2011; Herschbach, 2012; Michael and Overgaard, 2012; Overgaard and Michael, 2015).

Following Froese and Di Paolo (2011) and Froese et al. (2014a) then introduced a modification to the setup in order to make the task explicitly cooperative. First, participants are asked to cooperate and help each other find their avatars. Second, instead of a single long session in which participants can click without limits, the design is switched to 10–15 1-min long sessions, during which each participant is allowed a single click. Together, the two participants form a team in a tournament, playing against the other pairs of participants passing the experiment. The number of accurate and inaccurate clicks lets experimenters assign a post-experiment score to each team, and declare which pair of participants wins the tournament. Finally, experimenters introduce a questionnaire concerning each participants' clarity of perception of the presence of each other, using the Perceptual Awareness Scale (PAS; Ramsøy and Overgaard, 2004; Sandberg et al., 2010). PAS ratings for each interaction session go from 1 to 4, answering the following question: “Please select a category to describe how clearly you experienced your partner at the time you clicked: (1) No experience, (2) Vague impression, (3) Almost clear experience, (4) Clear experience.”

Framing the task as cooperative and making clicks a scarce resource led participants to spontaneously develop a new way of coordinating their behavior, namely turn-taking. Alice would oscillate around Bob while Bob remained static, and the roles would then be repeatedly swapped. This mutually regulated behavior first led participants to more accurately click on each other's avatars. Second, it confirmed the hypothesis that social cognition is partly constituted by social interaction: PAS ratings and turn-taking levels showed that participants developed first-person awareness of each other's presence during coordinated interactions.

### 2.4. Emergence of Coordination

Later analyses describe the way in which dyadic coordination emerges over successive trials in the form of turn-taking. This learning process is associated with an increase in social awareness as measured by PAS ratings, an increase in the proportion of trials in which both participants make successful clicks (Froese et al., 2014b), and an increase in the time spent with the other participant's avatar instead of the distractors (Hermans et al., 2020).

Inside trials, the emergence of social awareness has been associated with increased movement coordination as measured by cross-correlation and windowed cross-lagged regression between participants' movement time series. Stronger social awareness has also been linked to longer time lags in movement coordination, meaning that trials in which higher social awareness is achieved are likely to see participants coordinating and taking turns on a longer time scale than in trials with lower social awareness (Kojima et al., 2017). The precise dynamics leading a participant to click have also been shown to alternate passive and active stimulation time frames: in the second preceding a high social awareness click, information flows mostly from the person about to be clicked on, toward the person about to click, and the pattern is reversed with increasing strength up to 10 s after the click. In other words, high social awareness at the moment of a successful click is not an achievement of the person developing the awareness, nor of the other participant on their own; it is again a dyadic achievement (Kojima et al., 2017).

### 2.5. Shared Acts

The combined results of this research show that the task given to the participants is most successfully solved when both participants enter together in a coordinated shared act: Alice will detect Bob if Bob explores Alice, which he will do if Alice explores Bob, and so on. While the capacity for this kind of shared act emerges gradually over the trials, the end result can be well described in the framework of partial and shared acts developed by the Linguistic Bodies approach (Di Paolo et al., 2018). Let us then take a first step in abstracting out the structure of the interactions that take place in this paradigm. When Alice encounters an object, her exploring it will constitute a partial act of oscillatory stimulation. If Alice is faced with the shadow or the static object, the stimulation she will then receive (or lack thereof) will not allow for stable turn-taking to emerge. If the object is in fact Bob's avatar, Bob may respond to the received stimulation by a stimulation whose characteristics (rhythm, timing, duration) may constitute it as an appropriate response to Alice's partial act. This would lead to stable interaction dynamics where participants take turns in exploring each other, with increasing levels of social awareness. Bob may also, however, not respond appropriately or not respond at all, in which case Alice's partial act will be left unanswered, and the shared act fails.

In this context, the results presented so far indicate that an answer to such a partial act will have higher chances of success if it imitates the stimulation received, allows enough time for the partial act to be made, and allows for stable turn-taking to settle in. At this point in the interaction, both participants' movements strongly depend on each other, shared action is continuously being entertained, and social awareness will emerge.

Recent work has shifted toward investigating how strongly shared this kind of act is or can be (Froese et al., 2020; Hermans et al., 2020), and how variability across people enables it or hinders it (Zapata-Fonseca et al., 2018, 2019). For instance, Hermans et al. (2020) introduce a new measure of subjective experience and show that it is stronger in cases in which both participants click successfully, compared to cases in which neither participant clicks successfully, or only one of them does.

Beyond joint success, Froese et al. (2020) explored the basis for such social awareness. On one side, this could be a simple coordination behavior which allows the pair to enter a region of the dyadic phase space which is otherwise not attainable (*weak genuine intersubjectivity*, in the terms of Froese et al., 2020). On the other side, it could be the result of an event that is in some strong sense shared across the two participants, and merely reflected in their individual experiences of each other (*strong genuine intersubjectivity*). Indeed, in data reanalyzed by Froese et al. (2020), over 21% of the joint success trials show participants clicking within 3 s of each other. In other words, not only do participants develop social awareness of each other, they do so nearly at the same time. Froese et al. (2020) show that short inter-click delays are associated with higher individual and joint success, but only indirectly associated with higher subjective experience (PAS) of the other participant, such that the question of a single experience shared across the two participants is not yet settled.

Taking a step back, and temporarily setting aside the question of the intensity of intersubjectivity, it should now be clear that the structure of opportunities in which participants find themselves is very reminiscent of situations that are well-studied by Game Theory. As we will see, engaging in cooperation also bears a cost for players, and reaching joint success can also be seen as the result of participants navigating an action-dependent cost-benefit landscape, both individually and collectively.

In what follows I will propose a description of the PCP in the language of standard discrete Game Theory, and explore how previous results and open questions are rendered in the Game Theory framing. The shared action structure, in particular, appears at different time scales in the PCP and cannot be explained using traditional Game Theory only. On the other hand, Conditional Game Theory and Team Rationality can both account for the shared action structure of PCP, making this feature a useful contact point with the Linguistic Bodies approach.

## 3. A Game-Theoretic Description of the PCP

### 3.1. Framing the Task

We use the social agency version of the PCP task, as introduced by Froese et al. (2014a), where participants are presented as being part of a team, asked to click on each other *and* help each other succeed in doing so, but are otherwise not informed of any strategy for coordinating or succeeding at the task. Let us now simplify this task so that it can be framed, first, in the language of Decision Theory, and second, in the language of Game Theory. As a participant explores the space with their avatar, each stimulation received signals an encounter with one of the three objects in the space: the static object, the shadow (recall that the participant is not aware of the shadowing behavior), or the other participant's avatar. With no additional knowledge of the task, prior probabilities for an encounter with each of these objects are initially 1/3, and participants need to find their partner given two limited resources: (i) exploration time, and (ii) a single click. Each encounter can then be seen as two parallel decisions under uncertainty: whether or not to engage with the object at hand (if so, spending time to probe it and attempt to determine its nature), and whether or not to click.

We then make two important approximations. First, since a decision to click will formally end the primary task given by the experimenter (viz, clicking on the other), we set aside the click/no-click decision and focus on the decision about whether or not to engage with an encountered object, and if yes, how^3^. This keeps us free from too complex models where the uncertainties due to the two parallel tasks would interfere with each other, and lets us focus on the dynamics of the exploration-interaction task. At this point, the task can be more simply worded as “detect your partner in the space.” Our second approximation concerns the complexity of perceptual mistakes in this latter task. Indeed, a participant can make two types of errors in deciding whether an encountered object is their partner: thinking the object is their partner when it is not (type I error), and thinking it is not when in reality it is (type II error). Taking both these errors into account would require different probabilities for each error, such that decisions would be evaluated using two parallel and possibly conflicting criteria (one for each type of error to minimize). Instead, we set aside type I errors: our model assumes that when a participant believes they have found their partner, they are always right. In other words, a participant will never believe they have found their partner without actually having found them. Note that this in no way reduces the difficulty of the task, as the limited resource of exploration time is still present, and participants must still avoid type II errors: they may fail to perceive their partner if the interaction does not unfold well, or if the partner does not interact. At this point we can reword the task as “*find* your partner in the space,” which translates to a single continuous decision under uncertainty, which will now be possible to model: whether or not to engage with an encountered object, and if yes, how.

Finally, we discretize the situation. A perfect description of this task in the game theoretic framework would require us to take into account (i) the fact that the space of available decisions is continuous (rendering it a *continuous game*), (ii) the fact that decisions are continuously taken over time (possibly requiring the theory of *differential games*), and (iii) the long term memory involved in each decision. Such an analysis is beyond the scope of this paper, and we instead discretize each encounter in the following way. First, we reduce the timing of participants' choices to repeated discrete decision moments. Second, we approximate the space of possible strategies (which involve all variations between leaving, sensing, and actively interacting) to two options: (i) leaving or passively sensing (waiting to see if the other object explores my avatar), or (ii) interacting actively. In broad terms, these strategies are equivalent to (i) engage less (and save time for later encounters), (ii) engage more (and invest time).

### 3.2. Decision Theory Is Insufficient

A first, naive approach to this task would model it as a parametric exploration-exploitation trade-off decision. Given an unknown object, we denote the ordinal costs of interacting with it as 1 and not interacting as 0, and the benefit of interacting as the probability *p* that this object is the other participant. Naturally, interactions in the immediate past with the object at hand will change the expected probability that this object is the other participant. One way of incorporating this is to estimate the benefit as the posterior probability given past interactions, $b\left(\text{past}\right)=p\left(x=\text{other}|\text{past}\right)=p\left(\text{past}|x=\text{other}\right)\frac{p\left(x=\text{other}\right)}{p\left(\text{past}\right)}$. This cost-benefit situation is summarized in Table 1.

**Table 1:** Simple framing of the PCP as a parametric decision problem.

	**Benefit**	**Cost**
Don't engage	0	0
Engage	*b*(past)	1

In this framing, a possible strategy would be similar to the idealized honey bee exploration-exploitation problem^4^: devise a method for exploring the space, and use a criteria to engage in interaction which should be monotonic with respect to the expected benefit of the interaction.

As the results presented in the previous section make clear, however, success is not a matter of individual decisions: what one participant does is constituted by what their partner does, a fact that can be made apparent in the simple approximation of *b* above. Using the case of turn-taking between Bob and Alice, we know that if Alice engages in a partial act, Bob may respond with more stimulation, such that *p*(stimulation received∈past|*x* = Bob) will be higher if Alice has engaged in stimulation in the past. In other words, incoming stimulation has a different meaning depending on whether Bob is interacting or not, and Bob will interact differently depending on whether Alice has engaged in interaction in the past or not. While interaction is required for participants to reduce the uncertainty concerning an object encountered, it comes at the cost of time. The main question in this task, then, is when to interact, knowing that the outcome essentially depends on one's partner.

It is clear that this situation is not captured by Decision Theory, in which decisions and payoffs do not depend on the actions of other participants. Here, each participant's payoff depends on what the other participant does, such that a game theoretic description of the situation is warranted.

### 3.3. Modeling an Encounter

While still not formally representing a game, Table 2 provides a first representation of the partner-dependent choices faced by a participant, say Alice, if the nature and behavior of the object encountered were known.

**Table 2:** Choices faced by Alice, with corresponding payoffs and costs, were all the information available.

	**Object of encounter**				**Cost**

	**Static**	**Shadow**	**Bob engaging**	**Bob engaging**	
			**less**	**more**	
Engage less	0	0	1	2	0
Engage more	0	0	1	3	1

The numbers in the table represent the decision cost and ordinal preferences over the outcomes associated with each decision, given the nature of the object encountered, and in the case of an encounter with Bob, given Bob's strategy. When encountering the static object or the shadow, neither leaving nor engaging with it leads Alice to immediately find Bob, such that all ordinal preferences for the related outcomes are 0. When encountering Bob, the situation depends on Bob's strategy. If he is playing an “engage less” strategy, we consider that there is a slight possibility for Alice to detect Bob. However, we do not tie this possibility to the dynamics of the encounter nor to the time spent in the encounter (since Bob does not engage in it), so the ordinal preferences for the outcome with both strategies in the presence of non-engaging Bob can be set to 1. If Bob is engaging more, there is a higher likelihood of detecting him in both cases, but more so if Alice also engages in the interaction. The ordinal preferences for the outcomes are therefore 2 and 3. Whichever the actual object of encounter, engaging more bears the cost of time, which we initially represent as an ordinal cost of 1, compared to gaining time when not engaging, which we represent as an ordinal cost of 0.

Of course, during a real encounter the nature of the object is unknown, such that benefits and costs need to be combined to represent the choice under uncertainty that participants are faced with. Let us model the probabilities of detecting the other participant given their interaction strategy, and introduce parameters for the dependencies between the probability of each outcome.

First, let us label the “engage less” strategy L, and the “engage more” strategy M. Now, when Alice encounters an object *which in reality* is Bob, the probabilities that Alice detects Bob are as follows^5^:

- $\rho_{LL}$, if both play L
- $\rho_{LM}$, if Alice plays L and Bob plays M
- $\rho_{ML}$, if Alice plays M and Bob plays L
- $\rho_{MM}$, if both play M

We also introduce α∈[0, 1], the variable controlling Bob's strategy choices in the game: α is the probability that Bob plays M, and 1−α the probability for him to play L. Then let $p_{L}$ be the probability of Alice finding Bob by playing L, during an encounter with an unknown object. Conversely, let $p_{M}$ be the probability of her finding Bob by playing M with an unknown object. Since the probability that the unknown object actually *is* Bob is $\frac{1}{3}$, we have:


(1) $$\begin{array}{rc} p_{L}\left(\alpha \right) & =\frac{1}{3}\left(\left(1-\alpha \right)\rho_{LL}+\alpha \rho_{LM}\right) \end{array}$$

(2) $$\begin{array}{rc} p_{M}\left(\alpha \right) & =\frac{1}{3}\left(\left(1-\alpha \right)\rho_{ML}+\alpha \rho_{MM}\right) \end{array}$$

Now, considering that engaging in interaction requires more time than not engaging in interaction, we are interested in comparing the probabilities of detecting the other participant with different strategies *at constant time cost*. Let us then introduce τ∈ℕ^*^, the ratio of time costs between engaging and not engaging in interaction: if sensing with L takes 1 s, sensing with M takes τ seconds^6^. To compare the two strategies at constant time cost, therefore, we look at the probability $P_{L}$ that Alice will detect Bob by playing L during τ seconds: Alice can detect Bob during the first second with probability $p_{L}$, and if not (probability $1-p_{L}$), then in a second encounter during the second, or a third encounter in the third second, and so on and so forth. Then the probabilities of Alice detecting Bob using each strategy *at constant time cost* are:


(3) $$\begin{array}{l} P_{L}=p_{L}+\left(1-p_{L}\right)p_{L}+{\left(1-p_{L}\right)}^{2}p_{L}+\cdots +{\left(1-p_{L}\right)}^{\tau -1}p_{L}\\ \;=p_{L}\overset{\tau -1}{\underset{i=0}{\sum }}{\left(1-p_{L}\right)}^{i} \end{array}$$

(4) $$\begin{array}{rc} P_{M} & =p_{M} \end{array}$$

Now bringing Equations (1) and (2) into Equations (3) and (4), we obtain the benefit *g* of playing M over playing L, at constant time cost, as a function of α and the detection probabilities $\rho_{LL}$, $\rho_{LM}$, $\rho_{ML}$, and $\rho_{MM}$:


(5) $$\begin{array}{l} g\left(\alpha ,\rho_{LL},\rho_{LM},\rho_{ML},\rho_{MM}\right)=P_{M}\left(\alpha ,\rho_{ML},\rho_{MM}\right)\\ -P_{L}\left(\alpha ,\rho_{LL},\rho_{LM}\right) \end{array}$$

In order to render the exploration of this system of five variables palatable, let us add some final simplifications:

- let $u=\rho_{LL}=\rho_{ML}$ represent the probability of Alice detecting Bob, whichever Alice's strategy, during an encounter with Bob playing L^7^; indeed, we can reasonably consider this probability to not depend on the duration of the interaction, since Bob's L strategy ensures there is indeed very little interaction, and trying to interact more time with him will not increase the probability of feeling him^8^.
- let $w=\frac{\rho_{MM}}{\rho_{LM}}$ represent Alice's gain in playing M compared to L, during an encounter with Bob playing M.

## 4. Games and Strategies in the PCP

### 4.1. Encounter as an Assurance Game

**Figure 3:**
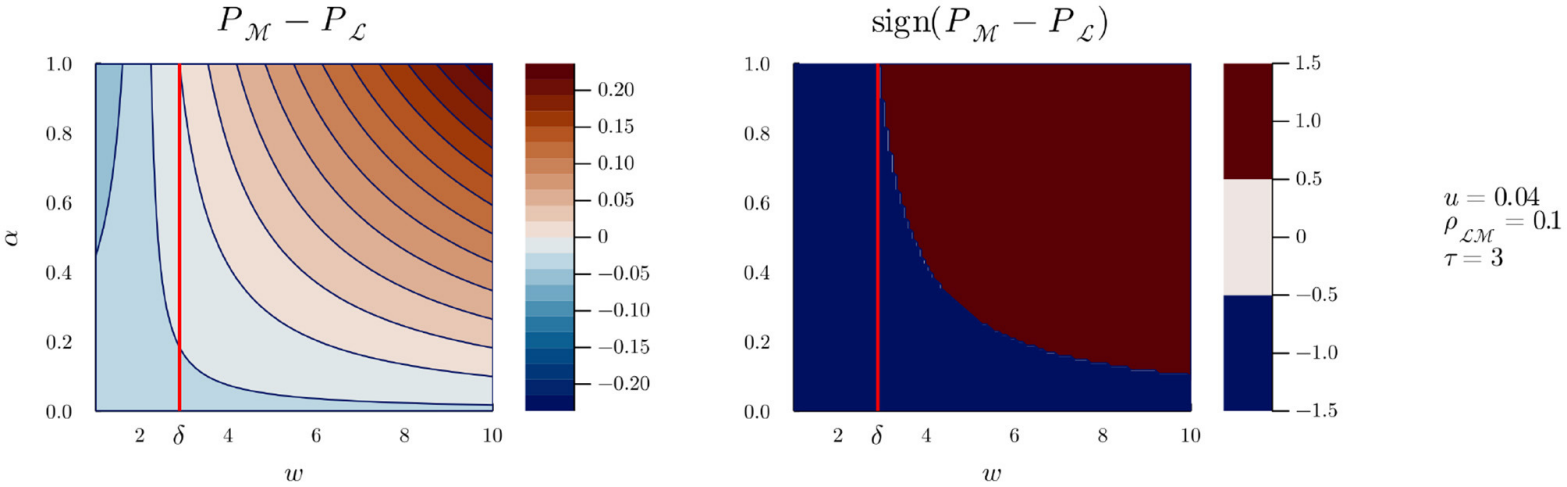
*g*(α, *w*) for *u* = 0.04, $\rho_{LM}=0.1$, and τ = 3.

Equipped with our model, and choosing values for *u* and τ^9^, we can represent the benefit of playing M vs. L during an encounter as a function of three variables: $g\left(\alpha ,\rho_{LM},w\right)$. The case *u* = 0.04 and τ = 3 (i.e., M costs three times more time than L), can be seen in Figure 3.

The left pane of Figure 3 shows the values of *g* at constant $\rho_{LM}=0.1$ (i.e., encountering Bob who plays M leads to a 0.1 probability of detection if Alice plays L), as a function of Bob's interaction strategy (α) and of the gain of playing more interaction if Bob also plays more interaction (*w*). The right pane represents the sign of *g*, as a function of the same variables. Colors closer to red (or simply dark red in the right pane) indicate higher values of *g*, that is, parameters for which Alice is more likely to detect Bob by playing M. Conversely, colors closer to blue (or simply dark blue in the right pane) indicate parameters for which Alice is less likely to detect Bob by playing M, that is she will be better off playing L. It is clear from both panes that for *w* < δ with τ>δ≈3^10^, Alice is better off always playing L, whereas for *w*>δ, there is a cutoff value of α above which Alice is better off playing M. As *w* grows, the cutoff value for α decreases, and Alice is better off playing M even if Bob has a relatively low α.

**Figure 4:**
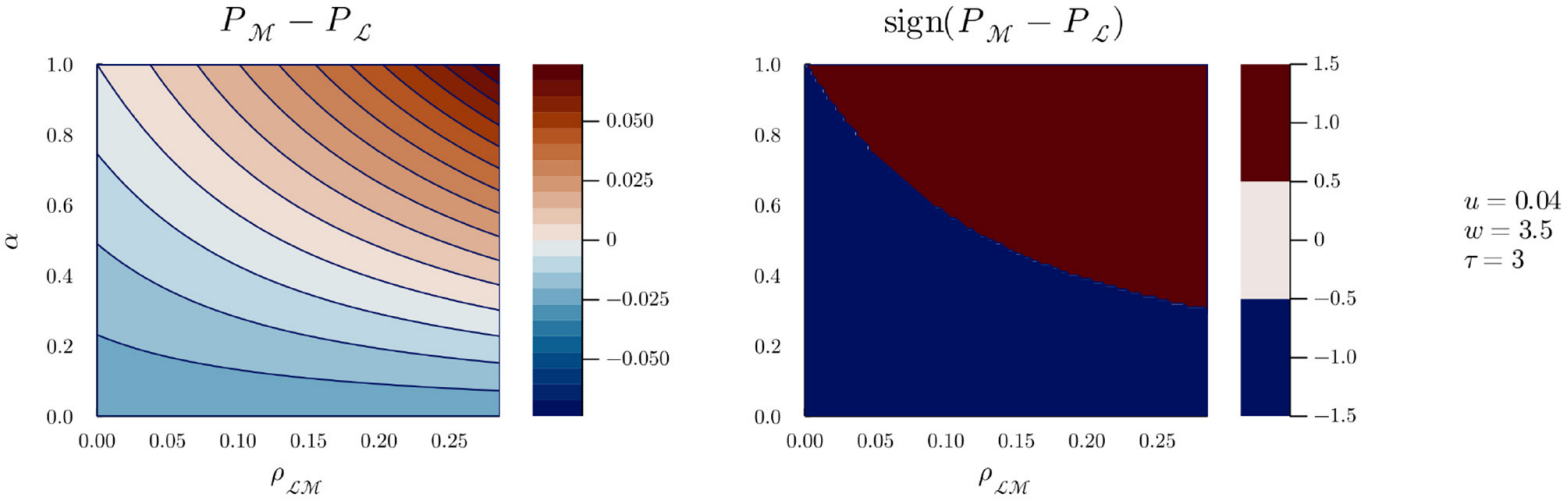
$g\left(\alpha ,\rho_{LM}\right)$ for *u* = 0.04, *w* = 3.5, and τ = 3.

**Figure 5:**
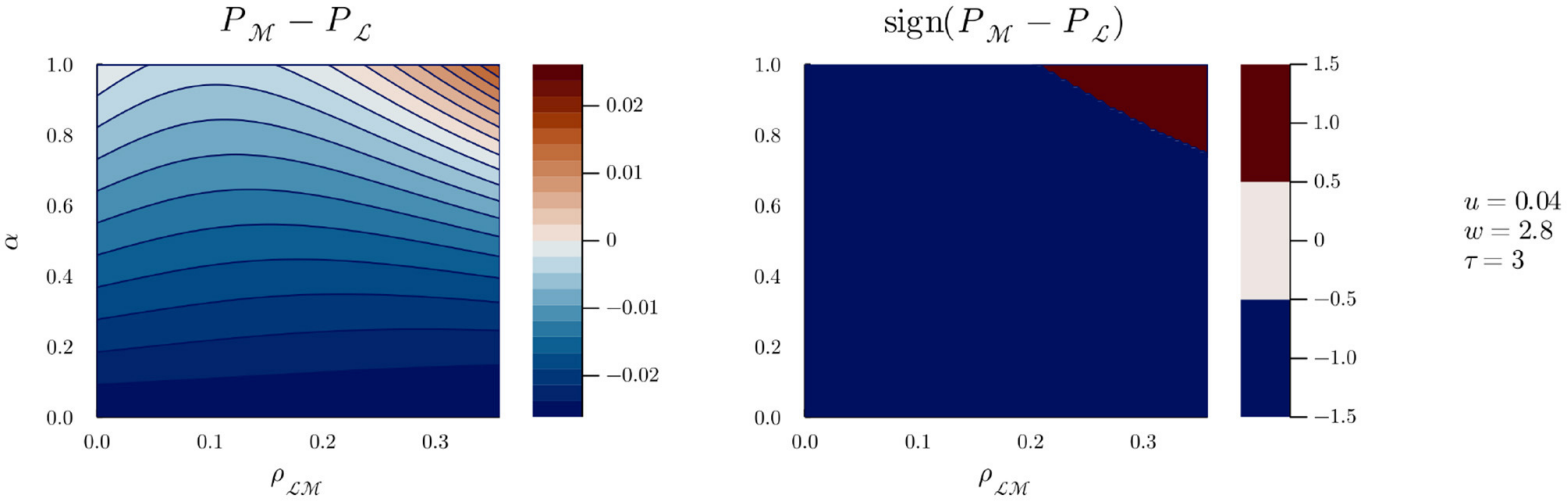
$g\left(\alpha ,\rho_{LM}\right)$ for *u* = 0.04, *w* = 2.8, and τ = 3.

If we now pick a value for *w*, say 3.5, we can inspect the evolution of *g* as $\rho_{LM}$ varies, as can be seen in Figure 4. A similar pattern can be seen: for all values of $\rho_{LM}$, there is a cutoff value of α over which Alice is more likely to detect Bob by playing M. The effect of a lower value for *w* can be seen in Figure 5: the range of values of α and $\rho_{LM}$ for which Alice is better off playing M is reduced, but does not disappear (it does, however, if *w* is further reduced). However, we can safely assume that when Bob plays M, the likelihood of detecting him grows with time at least equally whether Alice plays M or L; in other words, we can assume *w*≥δ. And without presupposing any result from previous work, we can further assume that when Alice plays M, she is involved in some active sensing, and the likelihood of detecting Bob (playing M) grows faster with time than when playing L. This is equivalent to stating that *w*>δ^11^, such that there will always exist a value of α above which Alice is better off playing M, and the situation represented in Figure 5 should not be possible.

**Figure 6:**
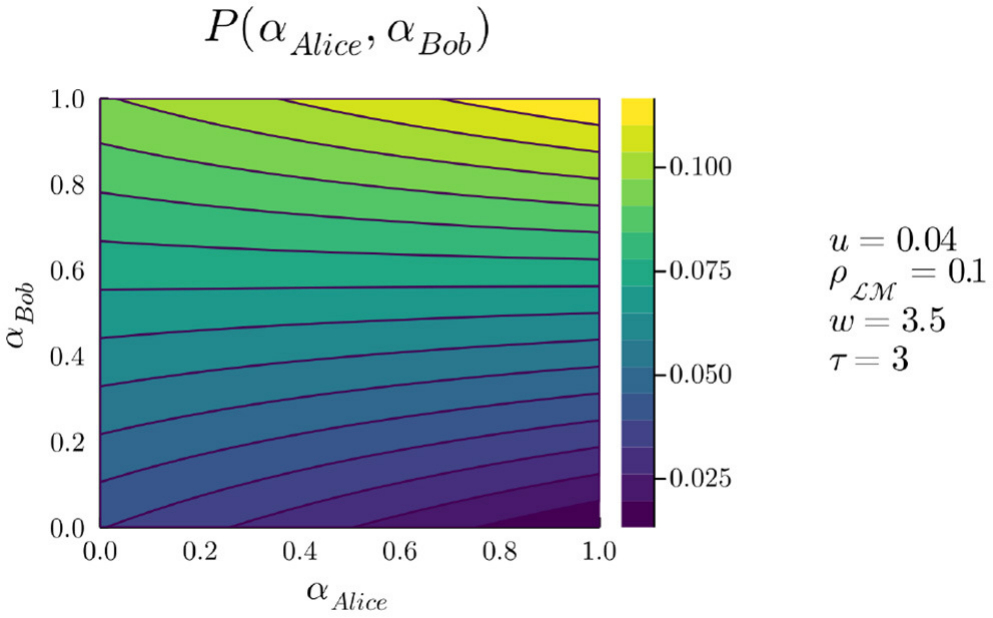
*P*(α_*Alice*_, α_*Bob*_) for *u* = 0.04, $\rho_{LM}=0.1$, *w* = 3.5, and τ = 3.

Given this knowledge, we can finally look at the expected benefits for Alice depending on the strategies she and Bob play. Figure 6 represents the probability that Alice will detect Bob at constant time cost, given fixed values for *u*, $\rho_{LM}$, *w*, and τ, as a function of α_*Alice*_ and α_*Bob*_:


(6) $$\begin{array}{r} P\left(\alpha_{Alice},\alpha_{Bob}\right)=\alpha_{Alice}P_{M}\left(\alpha_{Bob}\right)+\left(1-\alpha_{Alice}\right)P_{L}\left(\alpha_{Bob}\right) \end{array}$$

The plot first reflects what the previous figures indicated, when fixing α_*Bob*_ and inspecting Alice's options. For low values of α_*Bob*_, Alice is better off playing with low α_*Alice*_, that is favoring L. Conversely, for high values of α_*Bob*_, Alice is better off playing with high α_*Alice*_, that is favoring M. These patterns are maintained as long as *w*>δ, which we have seen is a reasonable assumption. Second, it is also clear that if both players maximize their α values, the likelihood of Alice feeling Bob at constant time cost is much higher than if both players minimize their α. The situation is of course symmetrical for Bob.

Let us now come back to binarized strategy options for both players, in terms of “high α” and “low α”. These two options correspond to favoring one or the other of M and L, though without committing to one or the other entirely. This setting corresponds to dicing Figure 6 into four quadrants. We see that Alice is worst off (dark blue) when Bob plays low α while Alice plays high α: Alice pays the cost of time by playing M while not getting any increase in probability of feeling Bob, since he plays L. A slightly better situation (dark green) is obtained when both play low α, that is, while Alice does not have a high probability of feeling Bob due to the two L strategies, she at least reduces time cost and therefore increases the possibility of feeling Bob in other encounters. A yet better situation (light green) occurs when Bob plays high α and Alice plays low α, and the best probability (light yellow) is obtained when both play high α. The values represented here are probabilities at constant time cost, which can be taken as payoffs for game actions; thus we can represent the ordinal preferences for each outcome, now including all costs incurred (incorporated in the computation of the probabilities), as shown in Table 3. The situation is identical for Bob (so symmetrical in the table), and the full game is represented in Table 4.

**Table 3:** Structure of the game faced by Alice.

		**Bob**	
		**Low α**	**High α**
Alice	Low α	1	2
	High α	0	3

**Table 4:** Structure of the game faced by both Alice and Bob.

		**Bob**	
		**Low α**	**High α**
Alice	Low α	1, 1	2, 0
	High α	0, 2	3, 3

This is not a Prisoner's Dilemma, but an Assurance game. If Bob plays low α (favoring L most of the time), Alice has the choice between playing high α and wasting time on encounters in which interaction is not reciprocated, or playing low α and moving from encounter to encounter, betting on the possibility that in one of them Bob will be detectable. Alice is better off following Bob's strategy: low α. If Bob plays high α, Alice can choose to passively receive Bob's stimulation (low α), or reciprocate interactions (high α) in which case detection is much more likely. She is better off again following Bob's strategy, in this case high α.

As the situation is identical for Bob, it follows that the game has two Nash Equilibriums, which are the two situations in which both players pick the same strategy.

### 4.2. Repeated Encounters

The model developed here partly sets aside the repeated nature of the game. First, encounters occur repeatedly during a single trial^12^, such that at this scale one can see the interaction as a repeated Assurance game which, in our approximation, ends for each player whenever they click.

More importantly, PCP experiments have repeated trials (going from 6 to 15 trials), over which participants learn about the space, the objects, and their interactions. Empirical results indicate players develop a stronger sensitivity and a more effective social interaction repertoire over time. In other words, over repeated trials interactions can become more effective, improving the signal-to-noise ratio and reducing uncertainty, which in the model is mainly represented by an increase in *w*. This becomes possible if players indeed engage in interactions, that is if they play high α. When played over repeated sessions therefore, the assurance game is reinforced: not only will players be more likely to find each other if they both play high α, but doing so from the start will even further increase the probability of detecting each other whenever they encounter each other, reducing the uncertainties and increasing the payoff associated with high α. Similarly to the session- and encounter-level games, if Alice plays this way but Bob doesn't, Alice will incur the cost of repeatedly playing high α without improvements in interactions (i.e., without increased *w*). A precise description of these dynamics requires more detailed modeling of the effects and costs related to learning over trials. While this is beyond the scope of this article, it seems likely that a similar structure could come to light, that is, another Assurance game could also describe the interaction at the scale of the experiment.

### 4.3. Summary of Results

Let us summarize the observations that can be made from this first description of PCP in the language of Game Theory.

We separated the PCP task into decisions about whether or not to click, and decisions about whether or not to interact. In order to focus on the decisions about interactive behavior, we set aside the decision about whether or not to click, and approximated the PCP as a situation in which participants never mistakenly think they are interacting with their partner (ignoring type I errors). In this approximation, the task is to “find” the other participant, type I errors (unsuccessful clicks) are ignored, and we focus on the relationship between type II errors (missed opportunity of a successful click) and interactions with an object that is not the other participant. This lets us concentrate on the structure of the “interact now or later” game which participants face, leaving further modeling of other aspects of the PCP for later work. We then assumed that the decisions participants are faced with in this game can be time-discretized into a series of “interact now vs. later” decisions, in which the option of interacting requires more immediate time investment than not interacting (or interacting less).

Next, we assumed the following approximations, which we take as a reasonable first approach to describing the PCP in the language of discrete Game Theory:

- the strategy of each player can be represented as a probability to interact or not interact (α), then later discretized to “low α” and “high α”
- interacting requires more time investment than not interacting, a relationship approximated with an integer factor (τ)

And we finally assumed the following relationships between the probabilities that can be defined given the approximations made thus far:

1. the probability of detecting a low α partner during an encounter with them, and whether interacting with them or not, is the same regardless of the time spent in the encounter ($\rho_{LL}=\rho_{ML}$)
2. when playing low α, the probability of detecting a high α partner is higher than the probability of detecting a low α partner ($\rho_{LM}>\rho_{LL}$)^13^
3. the probability of detecting a high α partner is higher when interacting than when not interacting [first because ${\tilde{\rho }}_{MM}\geq \rho_{LM}$, second because $P_{M}\left(1\right)>{\tilde{P}}_{m}\left(1\right)$, and these combine to yield *w*>δ]

These relationships are empirically supported. Previous work on the PCP has indeed shown that high perceptual awareness is associated with higher levels of turn-taking and behavior matching, is preceded by a period of passive stimulation, and is also associated with longer interaction times (Kojima et al., 2017, in particular Figures 6–8).

Under these approximations and assumptions, it appears that the “interact now vs. later” decisions have the structure of an Assurance game, by which players maximize their likelihood of finding the other if they play the same strategy, and more so if they both choose to favor more interaction. While previous experimental work has extensively shown that mutual high α is without doubt the best team-level strategy to find each other, the Assurance game discovered here adds new light to the PCP. First, it shows that a mutual low α strategy is also a Nash Equilibrium, a fact that is only apparent when one takes into account the time cost of interaction and the small but non-zero probability of finding the other in mutual non-interaction. Second, it shows that investing in interaction alone bears a higher cost than the mutual low α equilibrium, which accounts for the difficulty of the task: choosing between investing time now, with a higher win-lose uncertainty, or leaving that uncertainty for a later moment in the trial.

This result provides us with an empirical point of contact between Embodied Rationality and the two approaches capable of accounting for team behavior in the rationality-as-consistency tradition: Team Rationality and Conditional Game Theory.

## 5. Discussion

### 5.1. Standard Game Theory and Team Behavior

While the analysis process in Sections 3, 4 was couched in the language of Game Theory (consider the notions of choice, decision, strategy, or uncertainty), at this stage I am not suggesting that game-theoretic approaches are superior, or for that matter inferior, to other accounts of observed behaviors in PCP. Quite the contrary: given an empirical paradigm in which what seems like markedly human behaviors have been extensively documented, I aim to take the opportunity for conflicting accounts of human individual and group behavior to compete on a common ground. Two such approaches—Team Rationality and Conditional Game Theory—rely on the rationality-as-consistency framework and are therefore easy to assess in a game-theoretic framing. Besides, standard Game Theory itself *fails* to account for people's success in the Assurance game identified in PCP, such that the use of a game-theoretic description is really no more than a tool for rationality-as-consistency approaches to enter the debate. First then, let us see why standard Game Theory fails to account for PCP behavior, and set it aside.

The problem lies in the existence of several Nash Equilibriums in the Assurance game. Indeed, if the payoffs in a game are assumed to incorporate all the components of the preferences of players, and if that game then contains several Nash Equilibriums, standard Game Theory has no explanation for why an agent would prefer one equilibrium over another: by definition, all preferences have already been included in the derivation of the Nash Equilibriums. A good example of this problem appears in a recurrent yet misplaced criticism of the Prisoner's Dilemma game. As Ross (2021) describes it:

Many people find it incredible when a game theorist tells them that players designated with the honorific “rational” must choose in this game in such a way as to produce the outcome [(defect, defect)]. The explanation [of the “rational” choice] seems to require appeal to very strong forms of both descriptive and normative individualism.

Ross (2021) continues, citing Binmore (1994):

If players value the utility of a team they're part of over and above their more narrowly individualistic interests, then this should be represented in the payoffs associated with a game theoretic model of their choices.

In the case of the Prisoner's Dilemma, incorporating players' preferences for a more egalitarian outcome transforms the model into an Assurance game. Once this point is reached, standard Game Theory has no further tools to explain how agents choose one Nash Equilibrium over the other, even though the (Cooperate, Cooperate) equilibrium in the Assurance game is Pareto-optimal (Ross, 2021), and often chosen by people in practice. PCP is no exception here, and standard Game Theory is therefore ruled out as an account of well-documented behavior.

How, then, can convergence on the (Cooperate, Cooperate) equilibrium be accounted for? Both Team Rationality and Game Conditional Theory can answer this question. I will further propose an extension of ER, dubbed *Embodied Social Rationality*, which relies on the Linguistic Bodies approach to provide a third account of team behavior.

### 5.2. PCP Assurance Game Under Rationality-as-Consistency

Eschewing proposals that introduce components exogenous to rationality (such as heuristics or Schelling's notion of “focal points”), Sugden was the first to argue that accounting for team behavior should be done by extending the unit of agency. Thus, in cases where the existence of the team is already established, action are taken as part of a best-outcome plan for the team, subsuming the question of how to act depending on the action of one's partner (Sugden, 1993, p. 86):

To act as a member of the team is to act as a component of the team. It is to act on a concerted plan, doing one's allotted part in that plan without asking whether, taking other members' actions as given, one's own action is contributing toward the team's objective. … It must be sufficient for each member of the team that the plan itself is designed to achieve the team's objective: the objective will be achieved if everyone follows the plan.

Team Rationality removes each player's concern for possibly detrimental moves from their partner: a team-member who does not follow their part of the plan is *team-irrational*. In this framework, rationality is not a matter of optimizing for individual preferences (which can therefore vary freely without this resulting in theoretical deadlocks), but a matter of converging on mutually beneficial outcomes (Sugden, 2018, 2019). This form of reasoning can be illustrated in the PCP payoff landscape represented by Figure 6. If Alice is rational in the traditional, game-theoretic sense, she must consider Bob's strategy (α_*Bob*_) as fixed, and her movements on the payoff landscape are restricted to horizontal lines. If Alice and Bob are team-rational, they are free to move *together* on the payoff landscape. In both cases, Alice and Bob know that the best mutually beneficial outcome would result from high α_*Alice*_ combined with high α_*Bob*_. Yet in the first case, deciding under the assumption that the choice of the partner is fixed can prevent them from collectively reaching the best outcome, while in the second case they will each do their part in the concerted plan. The behavior of participants in the PCP Assurance game is thus understood using decision dynamics which span beyond individuals (Lecouteux, 2018). The role of a normative notion of individual preference, which has repeatedly been shown to conflict with empirical results (Infante et al., 2016), is also reduced.

Conditional Game Theory (Stirling, 2012, 2019) proposes a different account in which team agency is not needed, and for that matter need not exist. Instead, team behavior may emerge from the network of influences that agents' preferences exert on each other. Recall that a player's preferences are defined over the entire set of possible outcomes resulting from the actions of all players, such that conditioning on a player's preferences—instead of simply on their actions—substantially expands the dynamics possible in a Conditional Game Theory model. An analysis of the PCP Assurance game in this framework is beyond the scope of this discussion, yet the examples provided by Stirling and Tummolini (2018) and Hofmeyr and Ross (2019) for the Hi-Lo game suggest that the convergence of both participants on the “high α” behavior can be accounted for. This proposal has the additional benefit of applying to cases in which no team is established or payoffs are not as aligned as they are in the Hi-Lo and Assurance games. On the other hand, the way in which a player is influenced by another player's preferences may be a point of substantial variability across players. In particular, for an agent to obtain the actual (conditional) preferences of another agent influencing it, a fair amount of explicit communication or even computation may be required. This is in line with regular Game Theory's tradition of abstracting away from psychological details, but may render the conditional approach less applicable to the PCP case. By contrast, Team Rationality only requires players to be aware of the structure of the game, and consider themselves part of a team, both conditions which seem realized in the PCP.

### 5.3. The Evolution of Strategies

An important component of the enactive understanding of social agency in the PCP has so far not been addressed: the emergence and evolution of normative realms, that is, the horizon against which interactions are evaluated by participants. This notion encompasses both a participant's sensitivity to aspects of the interaction dynamics that take place and which they engage in, and their subjective valuation of such dynamics.

Contrary to the real Assurance game, strategy options in the PCP are open to change over time. Indeed, the existence of a strategy at a given point in time heavily depends on the history of interactions between the participants. After a small number of initial trials during which the framing from Sections 3, 4 is warranted, the PCP doesn't provide participants with fixed strategy options from which to choose. Instead, participants need to develop and stabilize their own set of dyadic interaction strategies. It is during this second phase of the experiment, once the initial strategies are being modified and tinkered with, that pairs of participants are able to develop social agency and genuinely perceive social presence. For instance, recent work shows that over successive trials the time spent with the other avatar increases (Hermans et al., 2020), along with an increase in the intensity of social awareness of the other (Froese et al., 2020), stronger levels of turn-taking and movement coordination, and longer interaction timescales (Kojima et al., 2017). The set of strategies to choose from at each encounter thus fluidly changes across trials as a result of the history of interactions in a pair.

The conceptual logic (if not the empirical unfolding) of this evolution is well explained by Participatory Sense-Making (De Jaegher and Di Paolo, 2007) and the Linguistic Bodies approach (Cuffari et al., 2015; Di Paolo et al., 2018). In a first step, two autonomous agents may maintain an initial contact without any pro-sociality, due to a stability related to each agent's sense-making process (i.e., each agent's regulation of self-environment interaction). In the PCP without click constraint, participants will come back on any object they sense, making the contact of two such agents stable over time. In a second step, a tension may emerge from the interference between the two agents' self-environment regulation processes. Indeed, at this point each agent's regulation process is active in an environment which includes the other agent, and therefore reacts differently to an environment from which the other agent is missing. In the PCP, this situation occurs when participants are constrained to a single click and the task is framed as cooperative: participants are more conservative with their click, and the cooperative framing may lead them to try and show themselves clearly to objects they encounter. This is the situation accounted for by the Assurance game.

Yet as agents actively explore different interaction dynamics, new co-regulation conventions emerge that solve the initial tension between the two agents' self-environment regulation processes. More elaborate stimulation of and reaction to the other participant's stimulation arises, marking the appearance of a co-regulation of the interaction. At this stage in the PCP, teams develop their own interaction conventions, associated with team-specific capacities for feeling each other, that is, a normative realm which sediments into a repertoire of shared acts: conventions which can be triggered by one participant (through a partial act) and call for an adequate response from the partner. Each shared act is a new form of meaningful interaction between partners, such that failing to respond adequately to a partial act can trigger new kinds of breakdown. Froese et al. (2014b) report the case of a participant feeling abandoned by their partner when an interaction was abruptly interrupted. On this view, such elaborate feelings result from the development and use of a repertoire of meaning-imbued shared acts, which constitute the new normative realm developed by the team.

The emergence of a repertoire of shared acts reconfigures the strategies that participants can use in each encounter: instead of remaining a fixed set, the strategies used by participants evolve over time, and are dependent on past interactions with the partner. This fundamental feature of the PCP, which underpins the emergence of a sense of social presence, cannot be explained by Conditional Game Theory or Team Rationality. Furthermore, abstracting the feature away for the purposes of models of collective behavior would negate the potential for evolution of choice sets as they emerge from agents' interactions themselves.

The time seems ripe, then, for a deeper comparison of Conditional Game Theory, Team Rationality, and the Linguistic Bodies approach. As the latter can account for fundamental features of the PCP which cannot be abstracted away by the former approaches, I believe that a second, deep comparison between the associated notions of rationality is also warranted: rationality-as-consistency, on one side, and Embodied Rationality, on the other. The analysis of the PCP presented in this paper shows that such comparisons are not only needed, but possible on empirical grounds.

## 6. Conclusion

In this article, I proposed a novel analysis of the PCP using the language of game theory. This analysis shows the existence of an Assurance game in the form of the “interact now-or-later” question that participants continuously need to solve. The existence of such a standard game in perceptual crossing sensorimotor interactions opened the door to comparing game theoretical approaches and the enactive theory of Participatory Sense-Making and Linguistic Bodies on two fronts. First, the capacity for participants to interactively solve the PCP Assurance game. Second, the evolution of choice landscapes resulting from the evolution of normative realms in the PCP. Finally, and most importantly, this work positions the PCP as an empirical meeting point between two radically different approaches to human interactions, namely, the economics tradition, interested in models of collective behavior such as markets, and the enactive approach.

## Data Availability Statement

The original contributions presented in the study are included in the article/Supplementary Material. The Julia Pluto notebook used to generate these figures and explore the model is available at the following url: https://gitlab.com/wehlutyk/2021-10-shared-embodied-rationality-paper-public/-/blob/main/encounter-game.jl. Further inquiries can be directed to the corresponding author.

## Author Contributions

The author confirms being the sole contributor of this work and has approved it for publication.

## Funding

This work was supported by OIST Innovative Technology Research - Proof of Concept Program.

## Conflict of Interest

The author declares that the research was conducted in the absence of any commercial or financial relationships that could be construed as a potential conflict of interest.

## Publisher's Note

All claims expressed in this article are solely those of the authors and do not necessarily represent those of their affiliated organizations, or those of the publisher, the editors and the reviewers. Any product that may be evaluated in this article, or claim that may be made by its manufacturer, is not guaranteed or endorsed by the publisher.

## Footnotes

^1^In short: Simon is a founding father of cognitivism; Kahneman and Tversky used “the axioms of logic and probability” as their normative benchmark; researchers in ecological rationality, while explicitly reducing reliance on internal models, “subscribe to Simon's computational program” in their understanding of “fast-and-frugal” heuristics (Petracca, 2021, p. 5).

^2^Or, for that matter, models built on the adaptive bounded rationality approaches compatible with a qualified computational metaphor of mind.

^3^Note that this is indeed an approximation, as participants sometimes explicitly click with uncertainty.

^4^A honey bee must decide whether to exploit a patch of flowers for which it knows the expected payoff, or explore the space to try and find a new patch of flowers which may or may not provide more payoff.

^5^Formally, these are the probabilities of avoiding a type II error.

^6^Thus, contrary to the Decision Theory model above, costs in this model have magnitudes instead of being ordinal.

^7^Recall that comparing L and M strategies *with an unknown object* and at constant time cost is done using $P_{L}$ and $P_{M}$, which will differ even though $\rho_{LL}=\rho_{ML}$. Indeed, playing L allows Alice to encounter several different objects (each of which may be Bob) in the same time cost as when encountering a single object and playing M.

^8^Introducing a factor $\frac{\rho_{ML}}{\rho_{LL}}>1$ does not qualitatively change the results, as long as it remains lower than a value *s*_*max*_ derived in the Supplementary Material. For $\rho_{LL}=0.04$ and τ = 3 as introduced in Section 4, we have *s*_*max*_≈0.98, that is $\rho_{ML,max}\approx 2.96\rho_{LL}$. See the Supplementary Material for more details.

^9^These variables have the least effect on *g*. See the Supplementary Material for more details.

^10^As δ is defined by $g\left(1,\rho_{LM},\delta \right)=0$, it is easy to derive that $\delta =\overset{\tau -1}{\underset{i=0}{\sum }}{\left(1-\frac{\rho_{LM}}{3}\right)}^{i}$. For $\rho_{LM}=0.1$ and τ = 3, we have δ≈2.901.

^11^Consider variations of $\rho_{MM}$, $p_{M}$, and $P_{M}$, marked with a tilde, which we use to represent the probability of detecting M-playing Bob by also playing M, but in the same time as when playing L. This lets us ask how probable it is to detect M-playing Bob by interacting with him during the same duration as it would take to not interact. We have ${\tilde{\rho }}_{MM}\geq \rho_{LM}$ and ${\tilde{p}}_{M}\left(1\right)=\frac{1}{3}{\tilde{\rho }}_{MM}$ (recall α = 1 since Bob plays M). Now our first assumption in the main text is that ${\tilde{P}}_{M}\left(1\right)$ follows the same form as $P_{L}\left(1\right)$ or a form that grows faster with τ. In other words, ${\tilde{P}}_{M}\left(1\right)\geq {\tilde{p}}_{M}\left(1\right)\overset{\tau -1}{\underset{i=0}{\sum }}{\left(1-{\tilde{p}}_{M}\left(1\right)\right)}^{i}$. Since $p_{M}\left(1\right)=P_{M}\left(1\right)\geq {\tilde{P}}_{M}\left(1\right)$, we have $p_{M}\left(1\right)\geq {\tilde{p}}_{M}\left(1\right)\overset{\tau -1}{\underset{i=0}{\sum }}{\left(1-{\tilde{p}}_{M}\left(1\right)\right)}^{i}$. Replacing with Equations (1) and (2) yields *w*≥δ. Our second assumption is that $P_{M}\left(1\right)>{\tilde{P}}_{M}\left(1\right)$, which in that case yields *w*>δ.

^12^Recent versions of the paradigm set the trial duration to 1 min (Froese et al., 2014a).

^13^Note that results sometimes still hold in the case where this is not satisfied, but we opted for assuming this conservative hypothesis to make the analysis palatable.
