# Supplementary material for: Embodied Rationality Through Game Theoretic Glasses: An Empirical Point of Contact
Source: Front Psychol. 2022 Apr 11;13:815691. doi: 10.3389/fpsyg.2022.815691 (PMC9035599; doi:10.3389/fpsyg.2022.815691)
Supplement: Supplementary file 1 [file Data_Sheet_1.pdf]

frontiers

Supplementary Material — Embodied rationality through game theoretic glasses: an empirical point of contact

1 INTRODUCTION

This supplementary material file shows the behaviour of the model introduced in the main text for different values of the parameters. The figures vary the parameters τ and u, maintained static in the main text, and introduce a parameter s to control the ratio $\frac{\rho_{\mathcal{ML}}}{\rho_{\mathcal{LL}}}$.

For more details, the Julia Pluto notebook used to generate these figures and explore the model is available at the following url: `https://gitlab.com/wehlutyk/2021-10-shared-embodied-rationality-/blob/main/encounter-game.jl`

2 DIFFERENT VALUES FOR τ

Varying the values of τ does not change the qualitative behaviour of the model, unless it results in $w < \delta$.

2.1 $\tau = 2$

$\tau = 2$ reinforces the behaviour demonstrated in the main text. See figures S1, S2, S3, and S4.

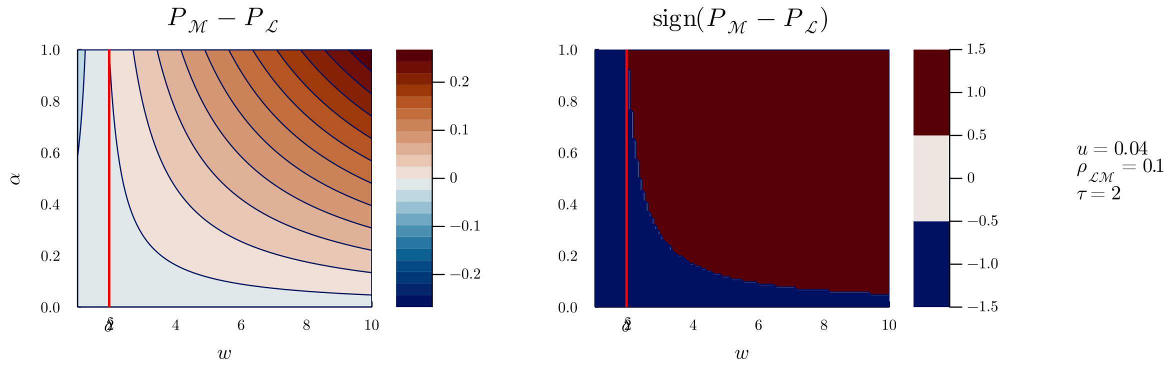

Figure S1. $g(\alpha, w)$ for $u = 0.04$, $\rho_{\mathcal{LM}} = 0.1$ and $\tau = 2$.

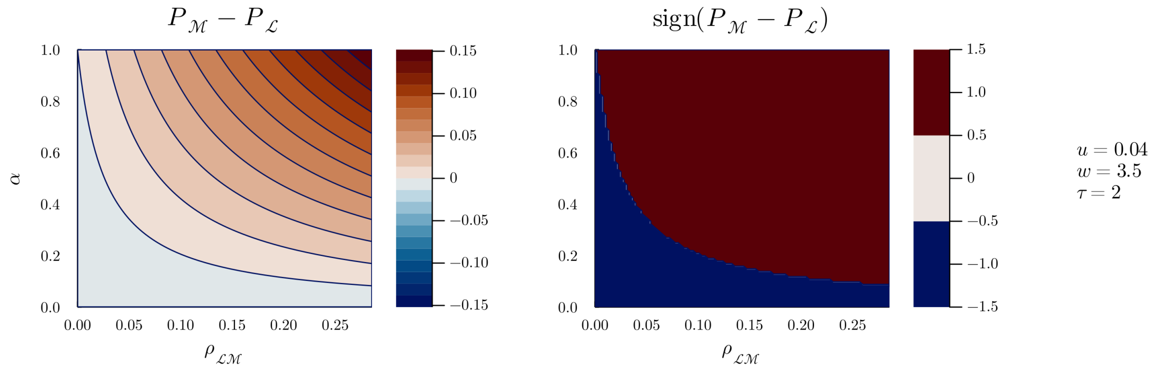

Figure S2. $g(\alpha, \rho_{\mathcal{LM}})$ for $u = 0.04$, $w = 3.5$ and $\tau = 2$.

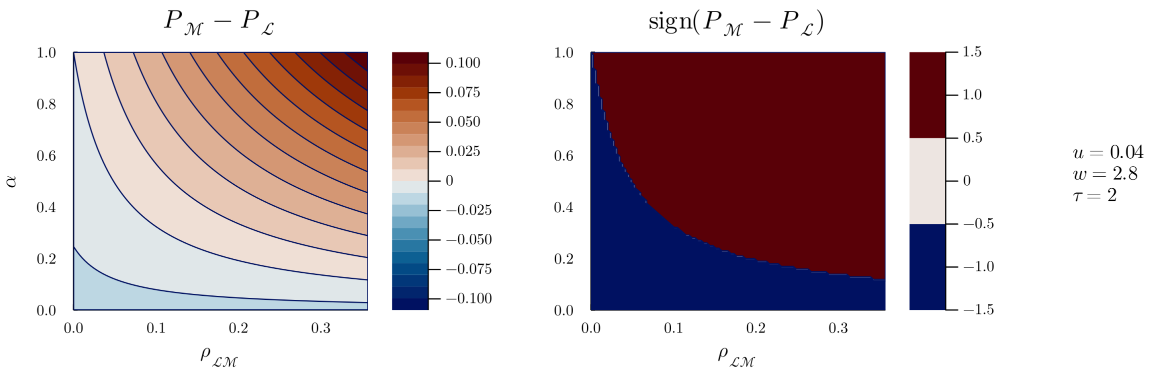

Figure S3. $g(\alpha, \rho_{\mathcal{LM}})$ for $u = 0.04$, $w = 2.8$ and $\tau = 2$.

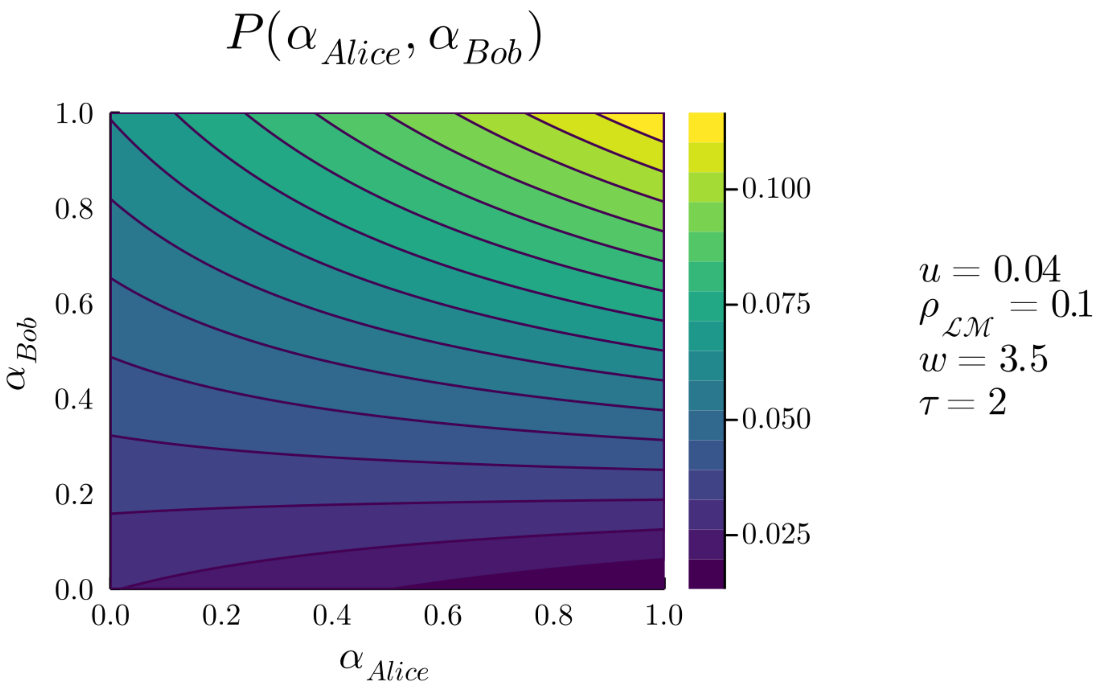

Figure S4. $P(\alpha_{Alice}, \alpha_{Bob})$ for $u = 0.04$, $\rho_{\mathcal{LM}} = 0.1$, $w = 3.5$ and $\tau = 2$.

2.2 $\tau = 4$

$\tau = 4$ results in $w < \delta$ (as now $\delta \approx 3.804$), such that the structure of the Assurance game is lost. See figures S5, S6, S7, and S8.

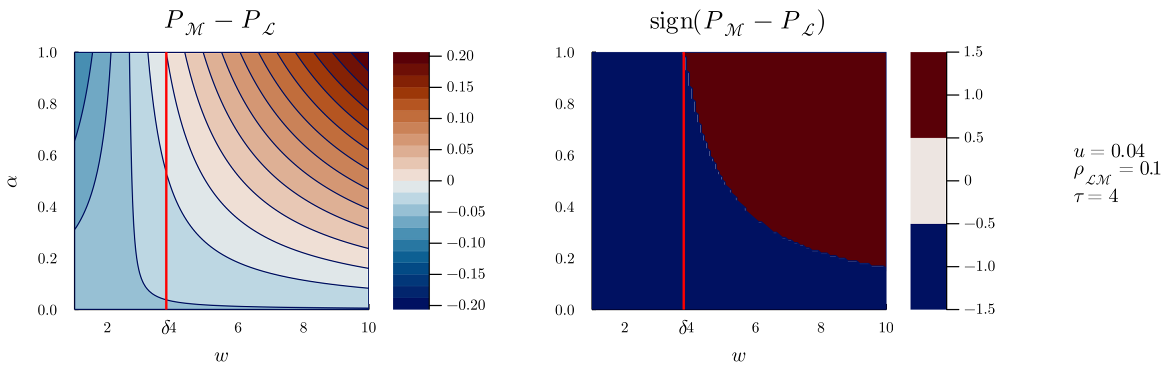

Figure S5. $g(\alpha, w)$ for $u = 0.04$, $\rho_{\mathcal{LM}} = 0.1$ and $\tau = 4$.

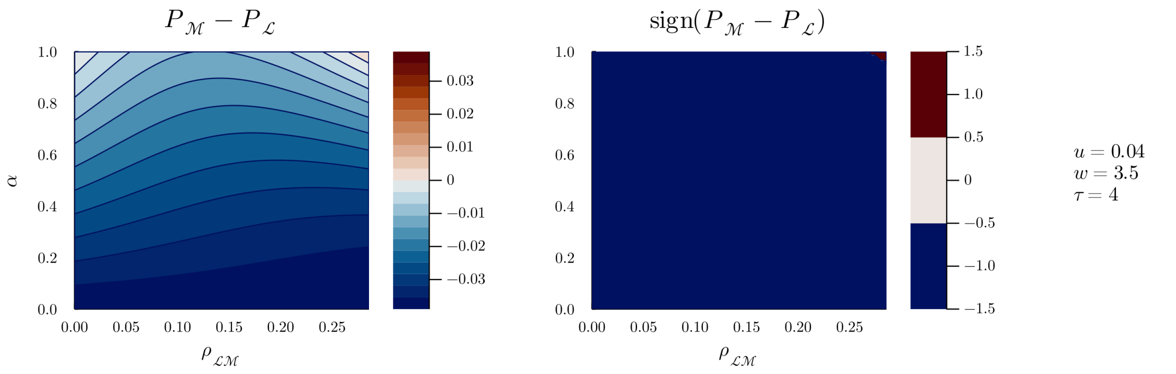

Figure S6. $g(\alpha, \rho_{\mathcal{LM}})$ for $u = 0.04$, $w = 3.5$ and $\tau = 4$.

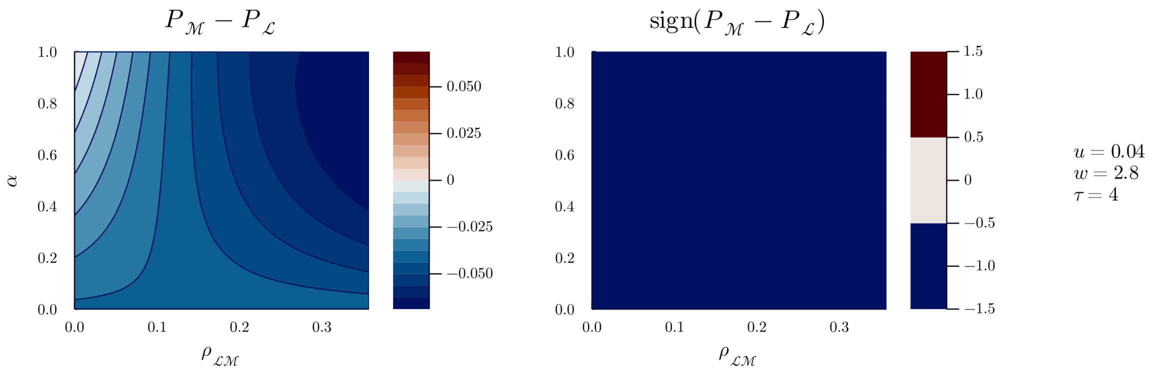

Figure S7. $g(\alpha, \rho_{\mathcal{LM}})$ for $u = 0.04$, $w = 2.8$ and $\tau = 4$.

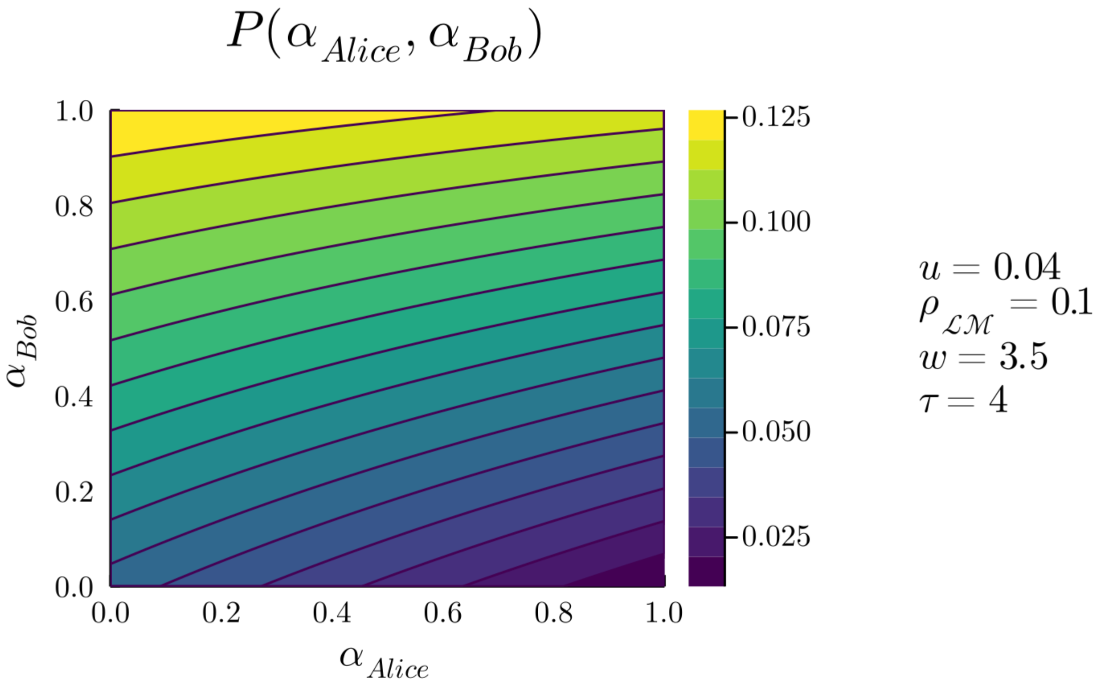

Figure S8. $P(\alpha_{Alice}, \alpha_{Bob})$ for $u = 0.04$, $\rho_{\mathcal{LM}} = 0.1$, $w = 3.5$ and $\tau = 4$.

2.3 $\tau = 4$ and $w = 4.0$

When correcting the value of w such that $w > \delta$, the Assurance game reappears. See figures S9 and S10.

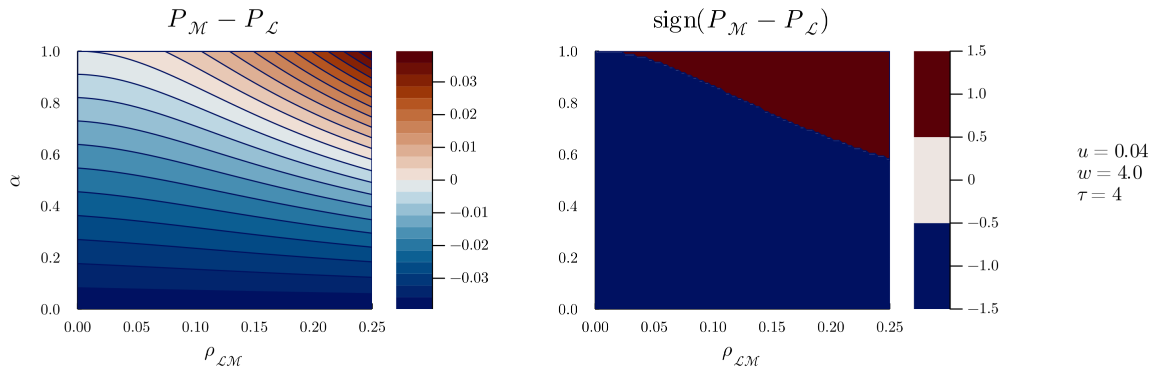

Figure S9. $g(\alpha, \rho_{\mathcal{LM}})$ for $u = 0.04$, $w = 4.0$ and $\tau = 4$.

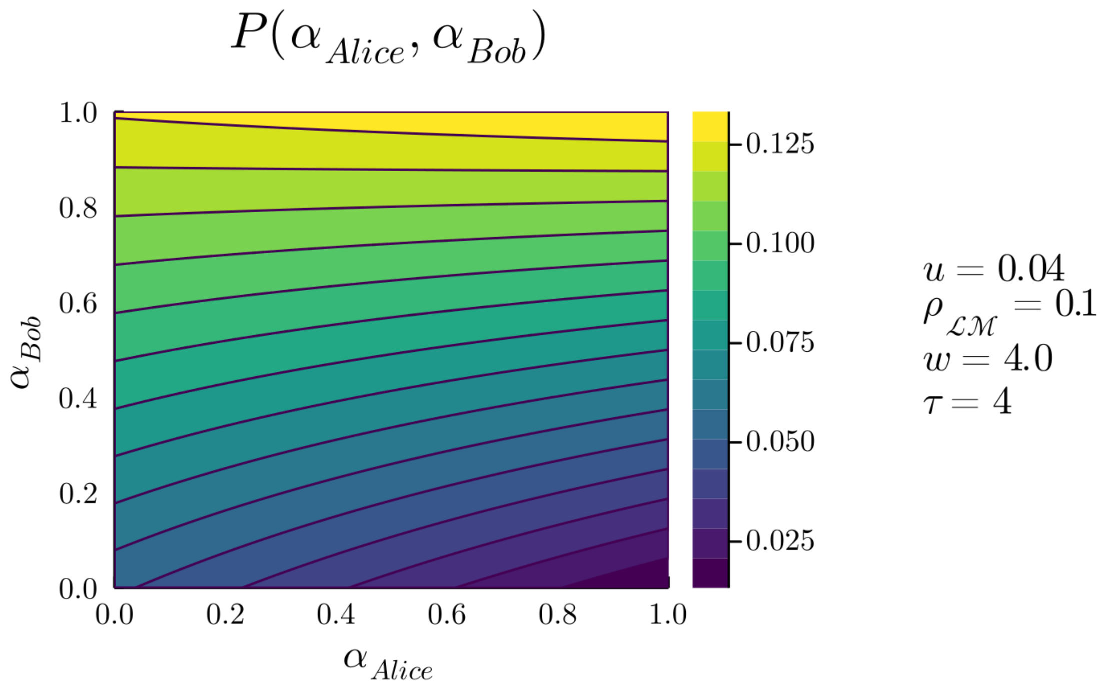

Figure S10. $P(\alpha_{Alice}, \alpha_{Bob})$ for $u = 0.04$, $\rho_{\mathcal{LM}} = 0.1$, $w = 4.0$ and $\tau = 4$.

3 DIFFERENT VALUES FOR U

Here we show the two extreme cases $u = 0$ and $u = \rho_{\mathcal{LM}}$.

3.1 $u = 0$

See figures S11, S12, S13 and S14.

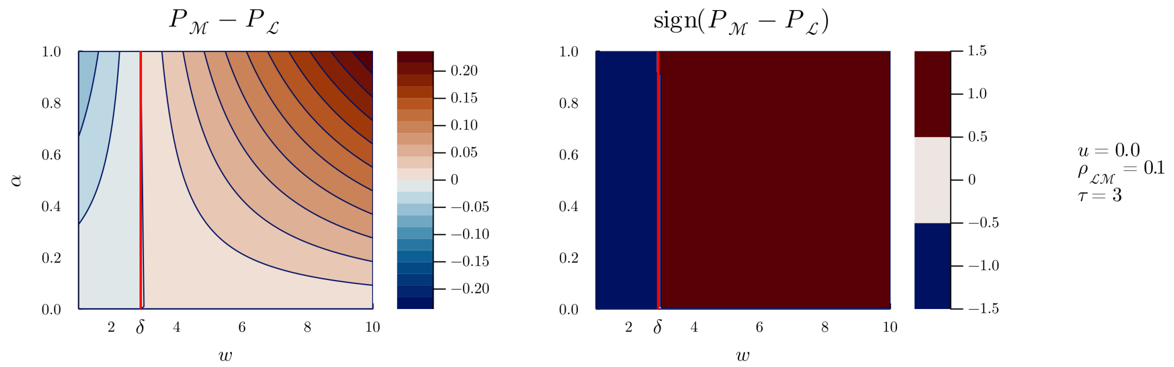

Figure S11. $g(\alpha, w)$ for $u = 0$, $\rho_{\mathcal{LM}} = 0.1$ and $\tau = 3$.

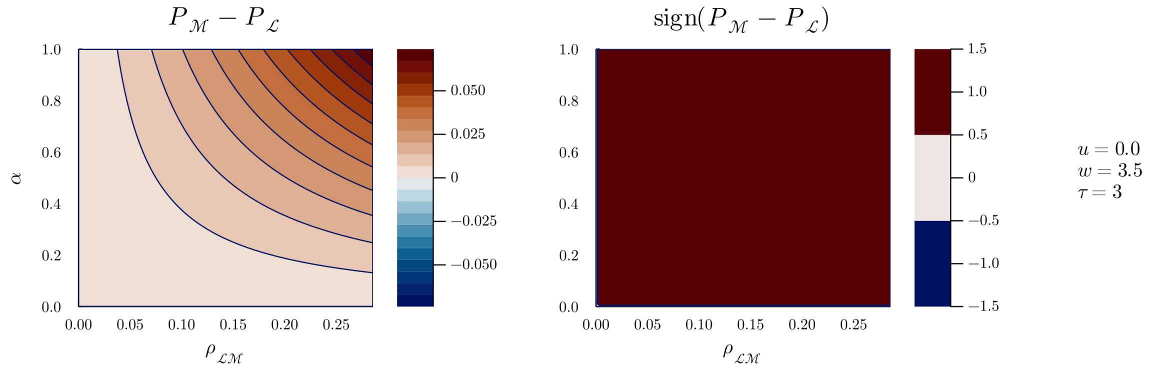

Figure S12. $g(\alpha, \rho_{\mathcal{LM}})$ for $u = 0$, $w = 3.5$ and $\tau = 3$.

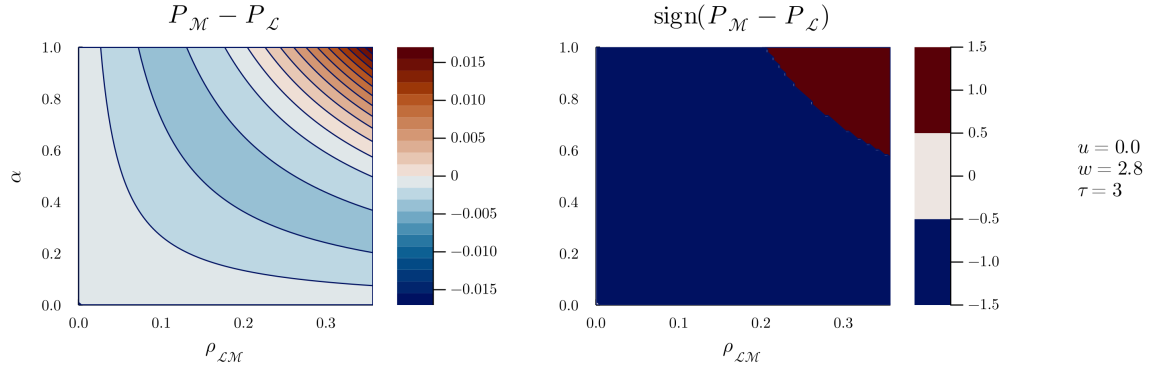

Figure S13. $g(\alpha, \rho_{\mathcal{LM}})$ for $u = 0$, $w = 2.8$ and $\tau = 3$.

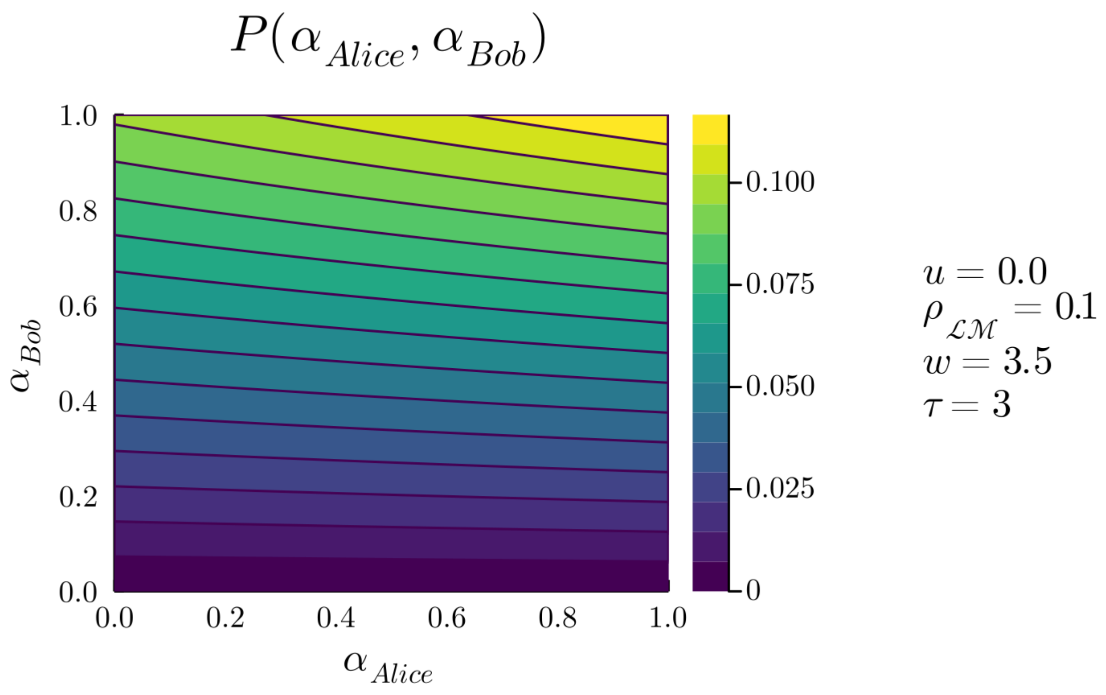

Figure S14. $P(\alpha_{Alice}, \alpha_{Bob})$ for $u = 0$, $\rho_{\mathcal{LM}} = 0.1$, $w = 3.5$ and $\tau = 3$.

3.2 $u = 0.1 = \rho_{\mathcal{LM}}$

See figures S15, S16, S17 and S18.

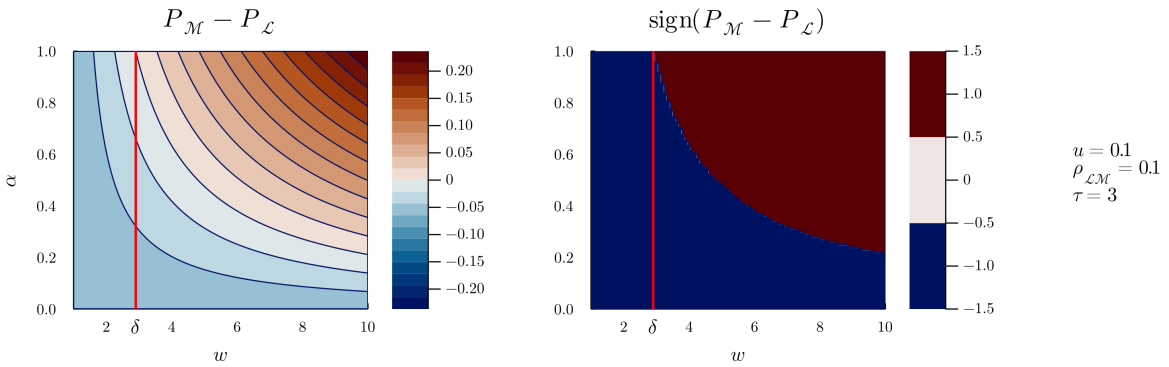

Figure S15. $g(\alpha, w)$ for $u = 0.1$, $\rho_{\mathcal{LM}} = 0.1$ and $\tau = 3$.

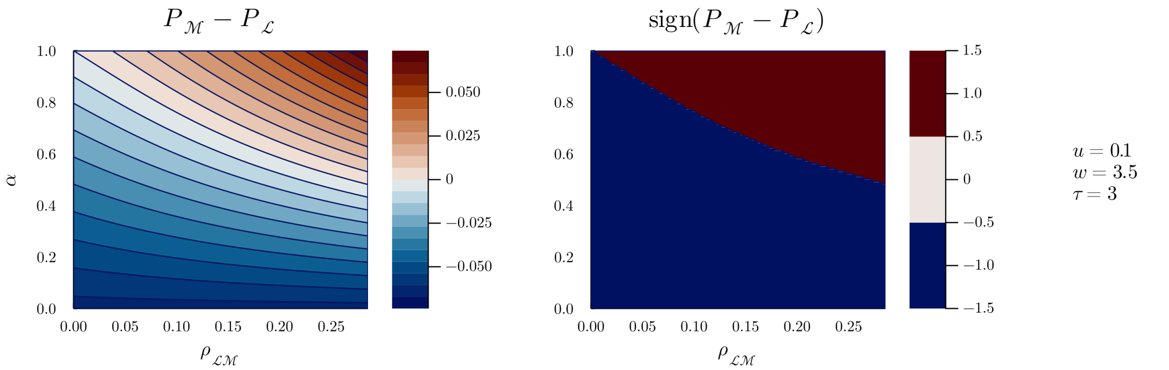

Figure S16. $g(\alpha, \rho_{\mathcal{LM}})$ for $u = 0.1$, $w = 3.5$ and $\tau = 3$.

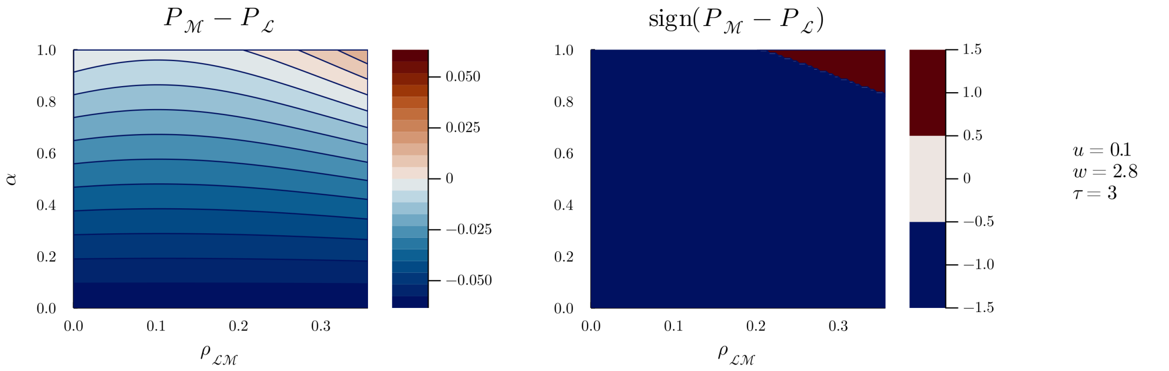

Figure S17. $g(\alpha, \rho_{\mathcal{LM}})$ for $u = 0.1$, $w = 2.8$ and $\tau = 3$.

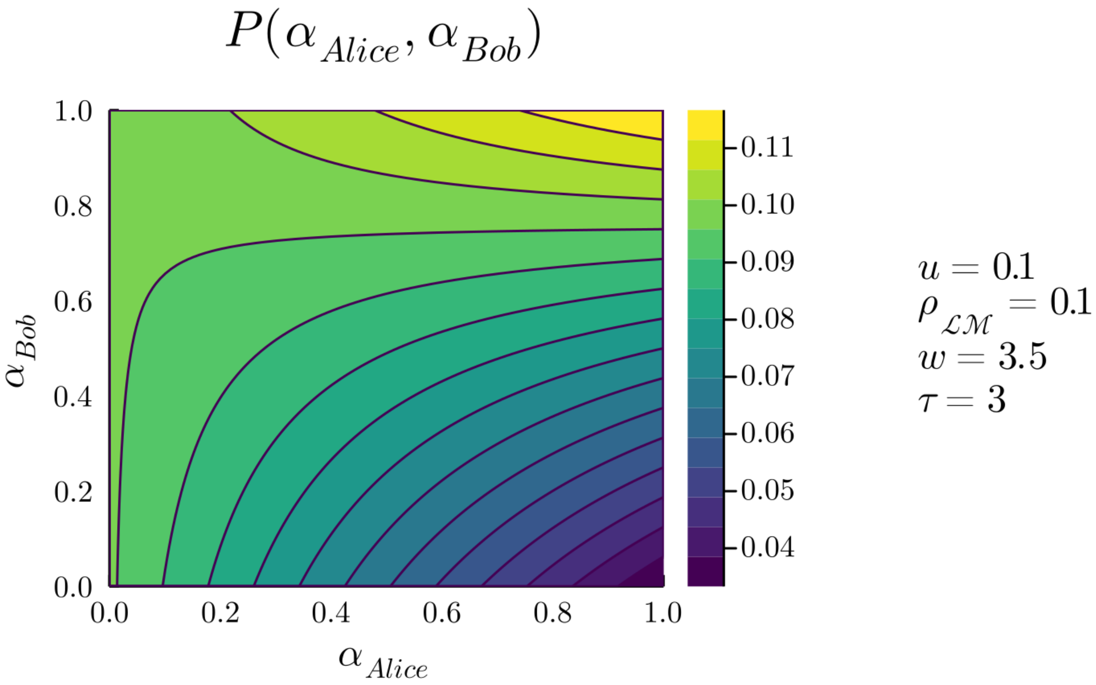

Figure S18. $P(\alpha_{Alice}, \alpha_{Bob})$ for $u = 0.1$, $\rho_{\mathcal{LM}} = 0.1$, $w = 3.5$ and $\tau = 3$.

4 VARYING $\frac{\rho_{\mathcal{ML}}}{\rho_{\mathcal{LL}}}$

Finally, to explore the relationship between $\rho_{\mathcal{ML}}$ and $\rho_{\mathcal{LL}}$, we redefine $u = \rho_{\mathcal{LL}}$ and introduce $s \in [0, s_{max}]$ such that

$$\frac{\rho_{\mathcal{ML}}}{\rho_{\mathcal{LL}}} = s\tau + 1 - s \tag{S1}$$

s_{max} is defined as the value of s which, for $\alpha_{Bob} = 0$ (Bob playing exclusively $\mathcal{L}$), cancels out the advantage of Alice playing $\mathcal{L}$ over playing $\mathcal{M}$. That is, s_{max} realises $P_{\mathcal{L}}(0) = P_{\mathcal{M}}(0)$. Replacing with equations 3 and 4 of the main text, then equations 1 and 2 of the main text, and finally equation S1 above, leads directly to

$$s_{max} = \frac{1}{\tau - 1}\left(\sum_{i=0}^{\tau-1}\left(1 - \frac{u}{3}\right)^{i} - 1\right)$$

For the values of the parameters used in the main text ($u = 0.04$, $\rho_{\mathcal{LM}} = 0.1$, $w = 3.5$, and $\tau = 3$), we have

$$s_{max} \approx 0.98$$
$$\rho_{\mathcal{ML},max} \approx 2.96\rho_{\mathcal{LL}}$$

Thus, when $s = 0$ we are in the situation described in the main text, that is $\rho_{\mathcal{ML}} = \rho_{\mathcal{LL}}$. As s grows, $\rho_{\mathcal{ML}}$ grows compared to $\rho_{\mathcal{LL}}$, to the extreme point at $s = s_{max}$ where $\rho_{\mathcal{ML}} = \rho_{\mathcal{ML},max}$, and the advantage of playing $\mathcal{L}$ over $\mathcal{M}$ when faced with $\alpha_{Bob} = 0$ is cancelled out.

4.1 $s = 0.25$

See figures S19, S20, S21 and S22.

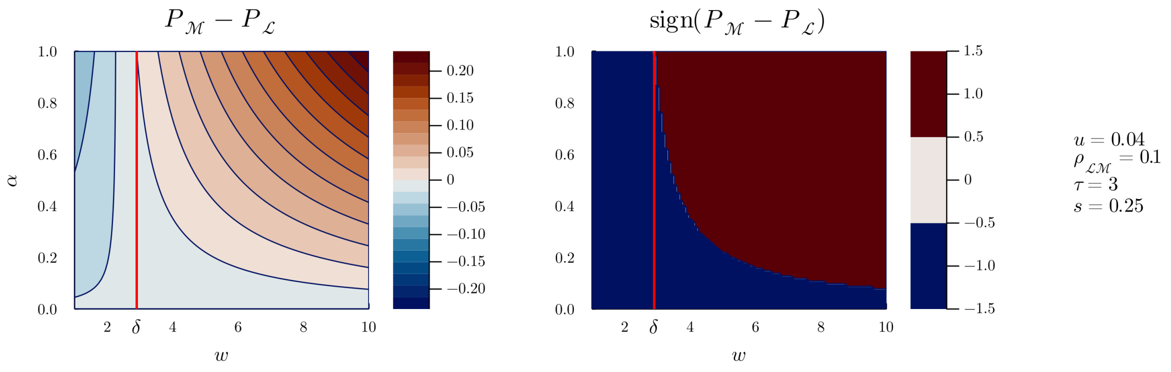

Figure S19. $g(\alpha, w)$ for $u = 0.04$, $\rho_{\mathcal{LM}} = 0.1$, $\tau = 3$ and $s = 0.25$.

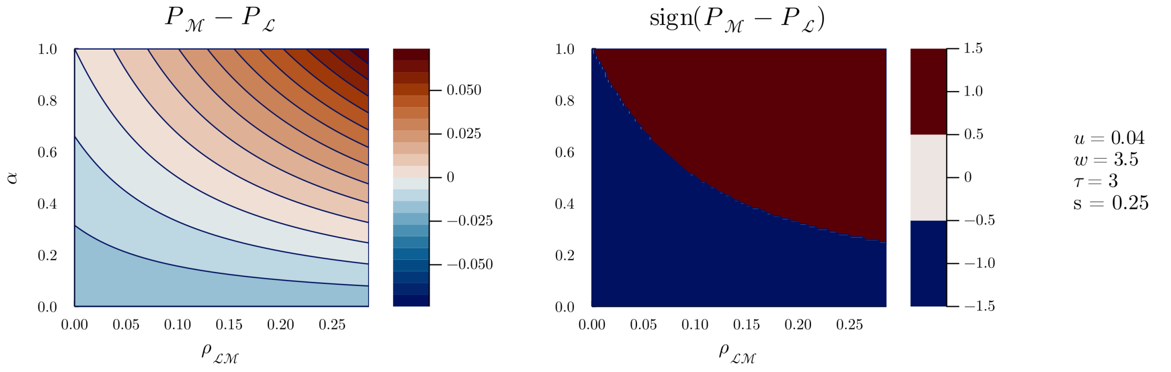

Figure S20. $g(\alpha, \rho_{\mathcal{LM}})$ for $u = 0.04$, $w = 3.5$, $\tau = 3$ and $s = 0.25$.

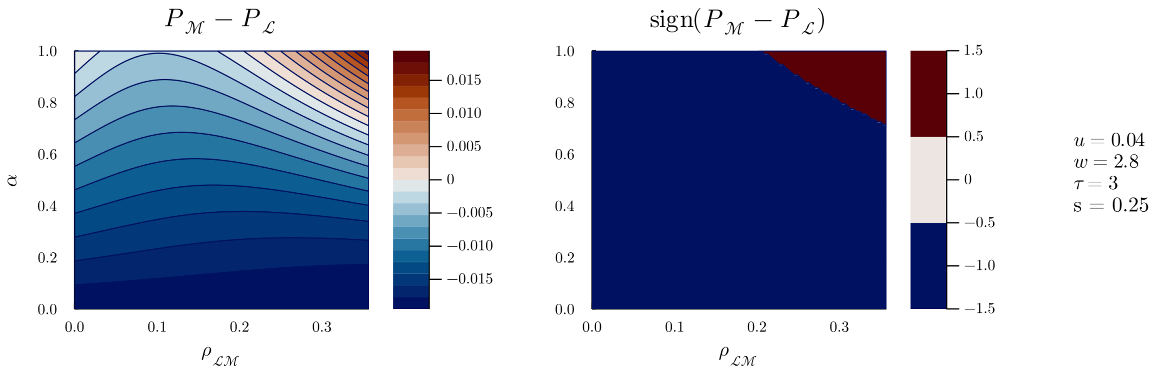

Figure S21. $g(\alpha, \rho_{\mathcal{LM}})$ for $u = 0.04$, $w = 2.8$, $\tau = 3$ and $s = 0.25$.

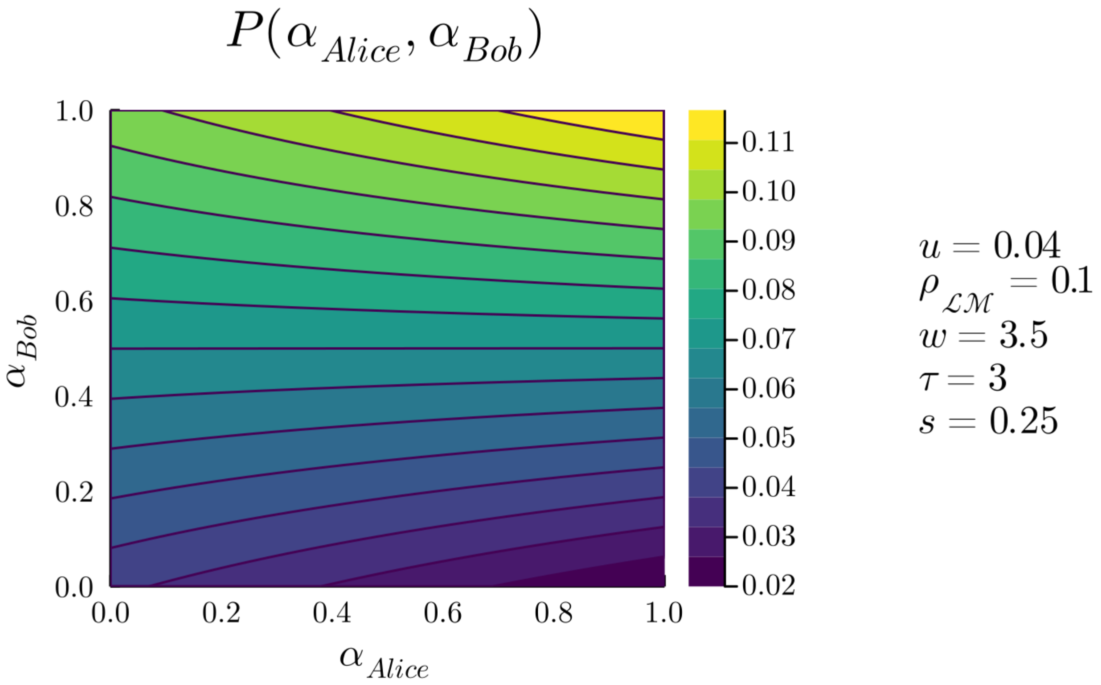

Figure S22. $P(\alpha_{Alice}, \alpha_{Bob})$ for $u = 0.04$, $\rho_{\mathcal{LM}} = 0.1$, $w = 3.5$, $\tau = 3$ and $s = 0.25$.

4.2 $s = 0.75$

See figures S23, S24, S25 and S26.

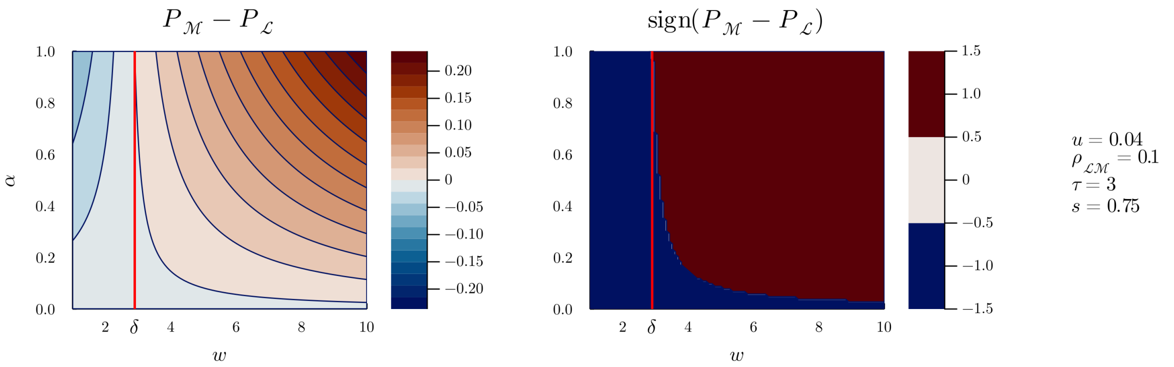

Figure S23. $g(\alpha, w)$ for $u = 0.04$, $\rho_{\mathcal{LM}} = 0.1$, $\tau = 3$ and $s = 0.75$.

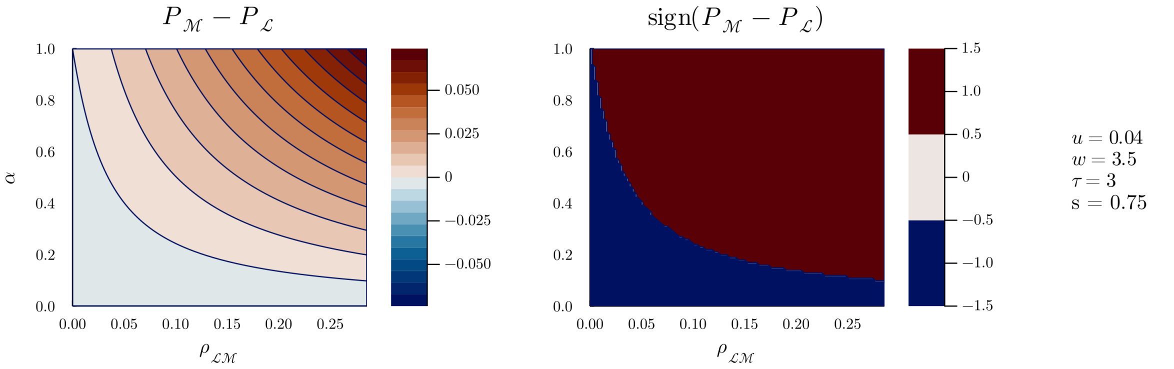

Figure S24. $g(\alpha, \rho_{\mathcal{LM}})$ for $u = 0.04$, $w = 3.5$, $\tau = 3$ and $s = 0.75$.

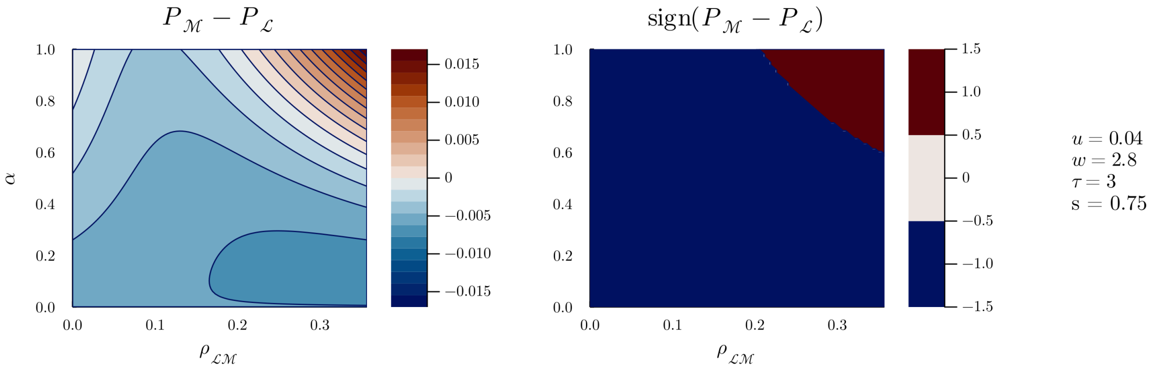

Figure S25. $g(\alpha, \rho_{\mathcal{LM}})$ for $u = 0.04$, $w = 2.8$, $\tau = 3$ and $s = 0.75$.

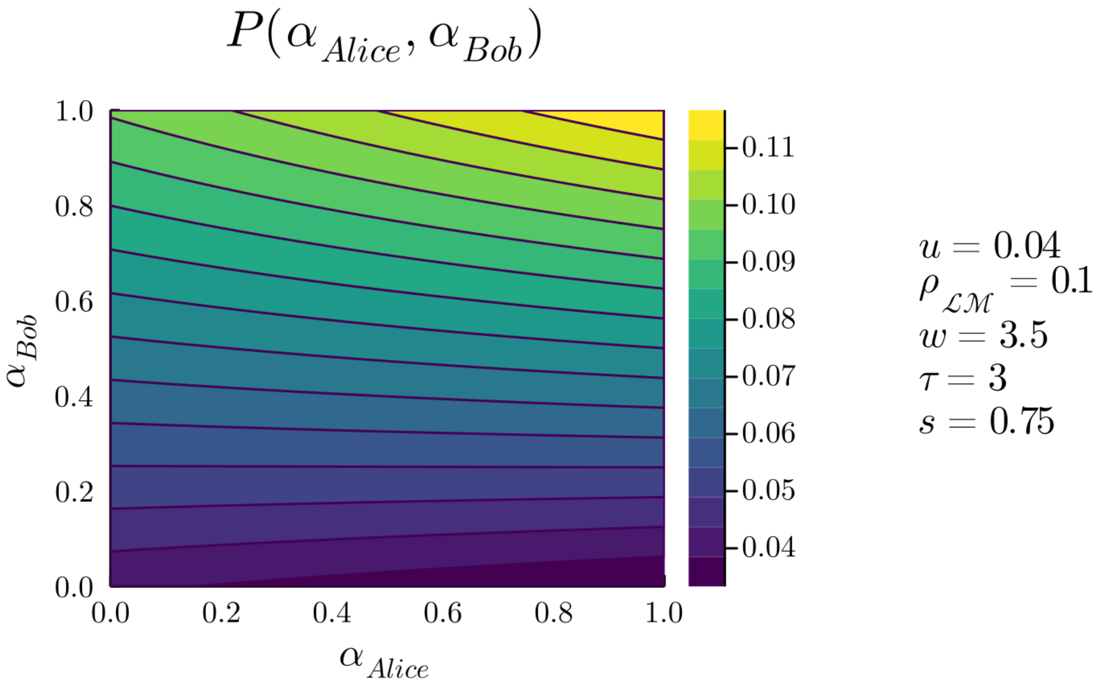

Figure S26. $P(\alpha_{Alice}, \alpha_{Bob})$ for $u = 0.04$, $\rho_{\mathcal{LM}} = 0.1$, $w = 3.5$, $\tau = 3$ and $s = 0.75$.

4.3 $s = 0.98 \approx s_{max}$

See figures S27, S28, S29 and S30.

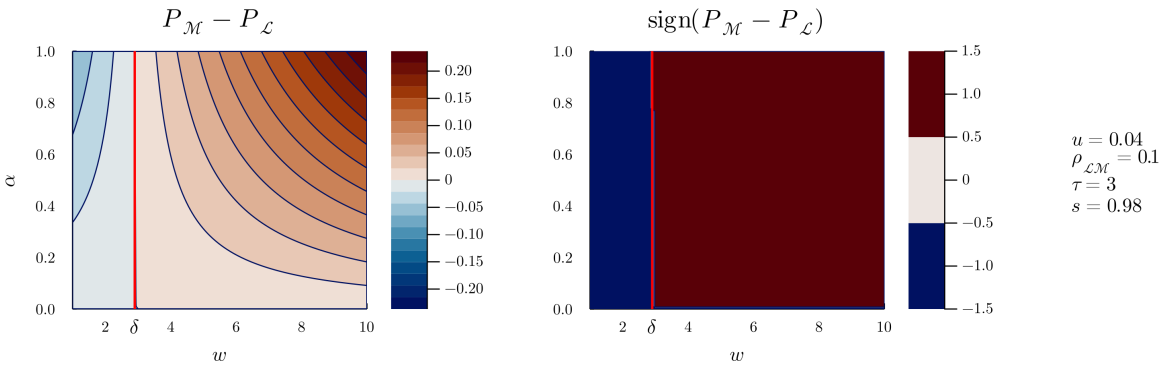

Figure S27. $g(\alpha, w)$ for $u = 0.04$, $\rho_{\mathcal{LM}} = 0.1$, $\tau = 3$ and $s = 0.98 \approx s_{max}$.

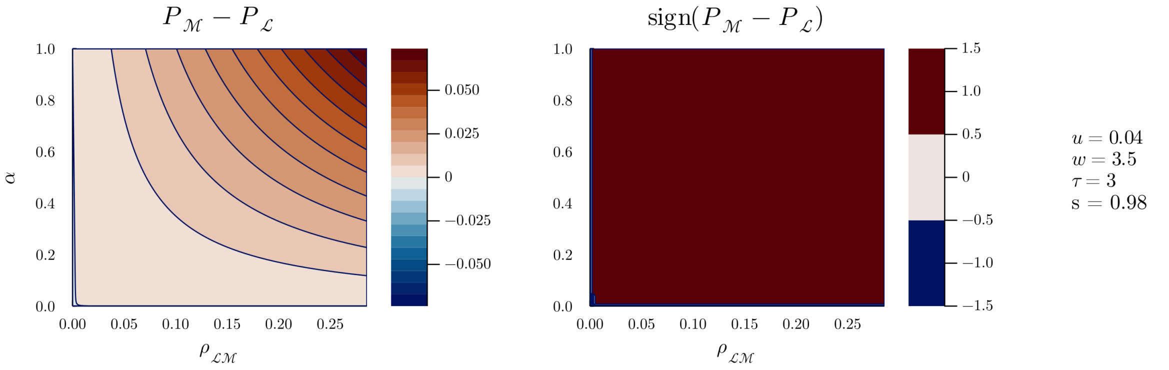

Figure S28. $g(\alpha, \rho_{\mathcal{LM}})$ for $u = 0.04$, $w = 3.5$, $\tau = 3$ and $s = 0.98 \approx s_{max}$.

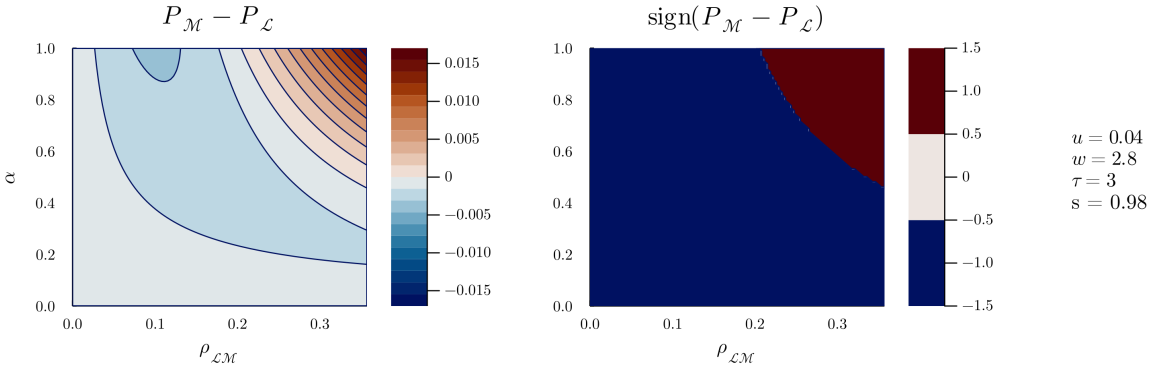

Figure S29. $g(\alpha, \rho_{\mathcal{LM}})$ for $u = 0.04$, $w = 2.8$, $\tau = 3$ and $s = 0.98 \approx s_{max}$.

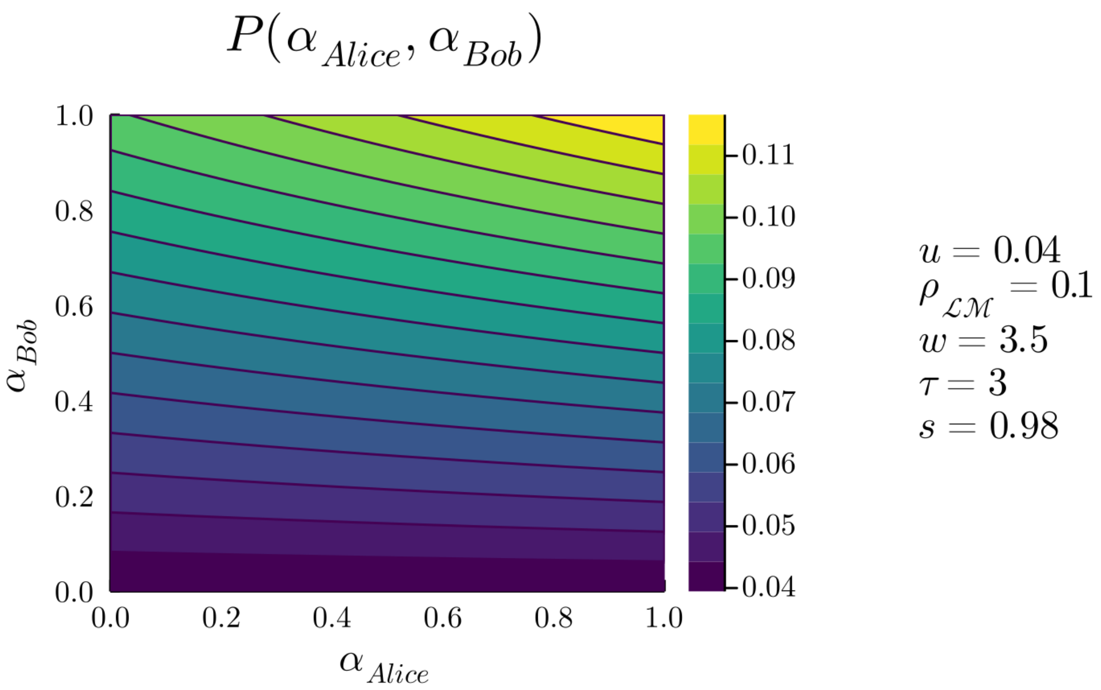

Figure S30. $P(\alpha_{Alice}, \alpha_{Bob})$ for $u = 0.04$, $\rho_{\mathcal{LM}} = 0.1$, $w = 3.5$, $\tau = 3$ and $s = 0.98 \approx s_{max}$.

4.4 $s = 1.0 > s_{max}$

See figures S31, S32, S33 and S34.

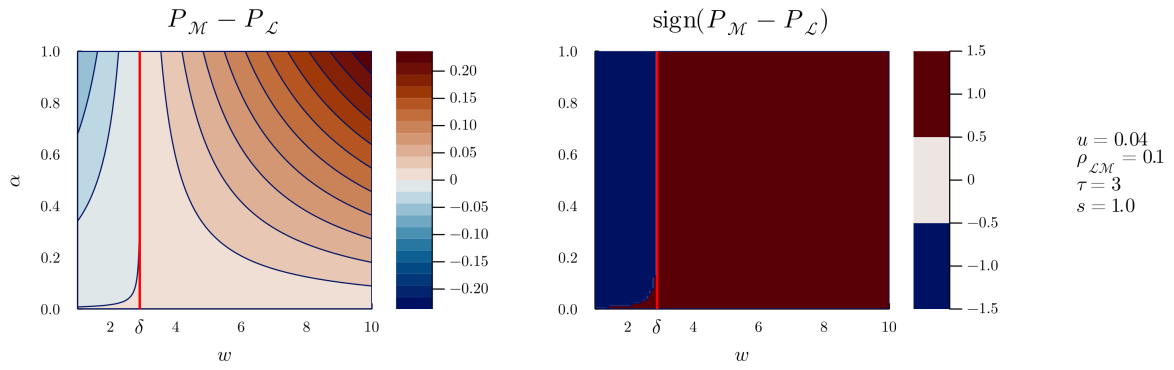

Figure S31. $g(\alpha, w)$ for $u = 0.04$, $\rho_{\mathcal{LM}} = 0.1$, $\tau = 3$ and $s = 1.0 > s_{max}$.

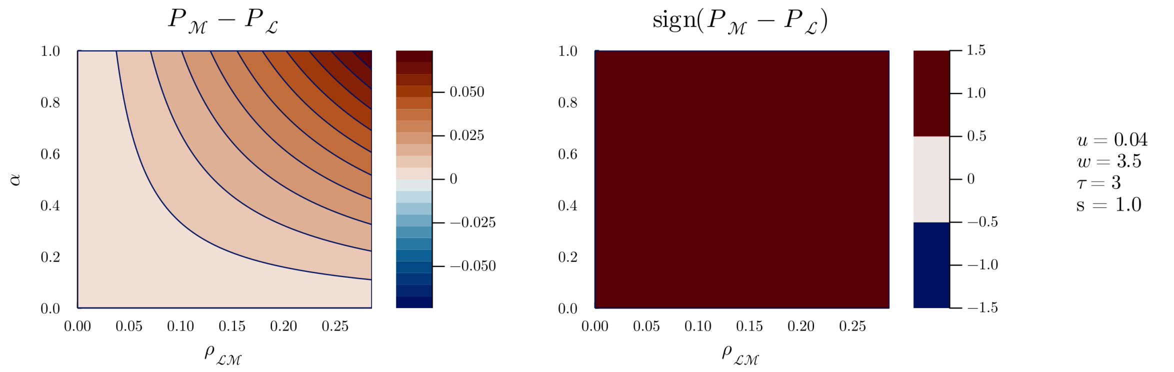

Figure S32. $g(\alpha, \rho_{\mathcal{LM}})$ for $u = 0.04$, $w = 3.5$, $\tau = 3$ and $s = 1.0 > s_{max}$.

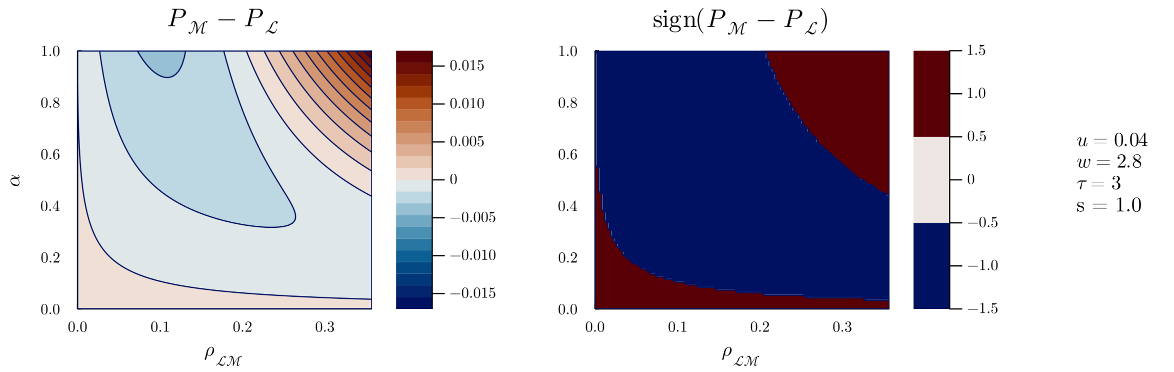

Figure S33. $g(\alpha, \rho_{\mathcal{LM}})$ for $u = 0.04$, $w = 2.8$, $\tau = 3$ and $s = 1.0 > s_{max}$.

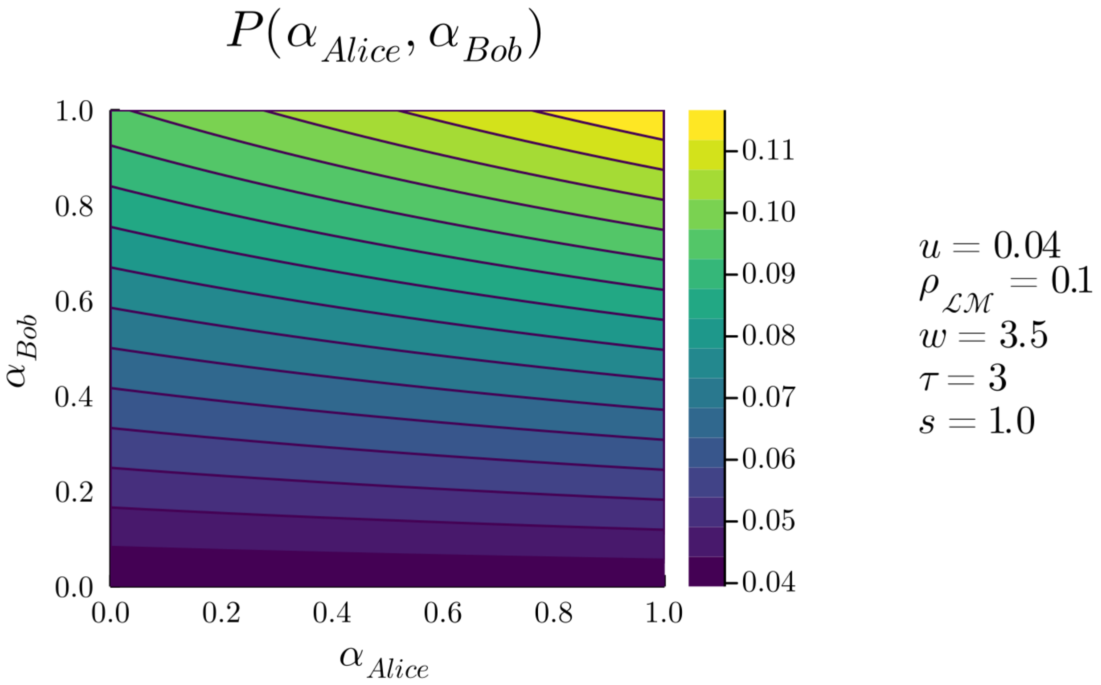

Figure S34. $P(\alpha_{Alice}, \alpha_{Bob})$ for $u = 0.04$, $\rho_{\mathcal{LM}} = 0.1$, $w = 3.5$, $\tau = 3$ and $s = 1.0 > s_{max}$.
